# TagF-mediated repression of bacterial type VI secretion systems involves a direct interaction with the cytoplasmic protein Fha

**DOI:** 10.1074/jbc.RA117.001618

**Published:** 2018-03-29

**Authors:** Jer-Sheng Lin, Panayiota Pissaridou, Hsin-Hui Wu, Ming-Daw Tsai, Alain Filloux, Erh-Min Lai

**Affiliations:** From the ‡ Institute of Plant and Microbial Biology and; the ¶ Institute of Biological Chemistry, Academia Sinica, Taipei 11529, Taiwan and; the § Medical Research Council Centre for Molecular Bacteriology and Infection, Department of Life Sciences, Imperial College London, London SW7 2AZ, United Kingdom

**Keywords:** bacterial genetics, gene regulation, protein secretion, protein phosphorylation, protein-protein interaction, Agrobacterium tumefaciens, antibacterial activity, post-translational regulation, Pseudomonas aeruginosa, type VI secretion system, TagF, PppA, Fha

## Abstract

The bacterial type VI secretion system (T6SS) delivers effectors into eukaryotic host cells or toxins into bacterial competitor for survival and fitness. The T6SS is positively regulated by the threonine phosphorylation pathway (TPP) and negatively by the T6SS-accessory protein TagF. Here, we studied the mechanisms underlying TagF-mediated T6SS repression in two distinct bacterial pathogens, *Agrobacterium tumefaciens* and *Pseudomonas aeruginosa.* We found that in *A. tumefaciens*, T6SS toxin secretion and T6SS-dependent antibacterial activity are suppressed by a two-domain chimeric protein consisting of TagF and PppA, a putative phosphatase. Remarkably, this TagF domain is sufficient to post-translationally repress the T6SS, and this inhibition is independent of TPP. This repression requires interaction with a cytoplasmic protein, Fha, critical for activating T6SS assembly. In *P. aeruginosa*, PppA and TagF are two distinct proteins that repress T6SS in TPP-dependent and -independent pathways, respectively. *P. aeruginosa* TagF interacts with Fha1, suggesting that formation of this complex represents a conserved TagF-mediated regulatory mechanism. Using TagF variants with substitutions of conserved amino acid residues at predicted protein–protein interaction interfaces, we uncovered evidence that the TagF–Fha interaction is critical for TagF-mediated T6SS repression in both bacteria. TagF inhibits T6SS without affecting T6SS protein abundance in *A. tumefaciens*, but TagF overexpression reduces the protein levels of all analyzed T6SS components in *P. aeruginosa*. Our results indicate that TagF interacts with Fha, which in turn could impact different stages of T6SS assembly in different bacteria, possibly reflecting an evolutionary divergence in T6SS control.

## Introduction

The type VI secretion system (T6SS)^4^ is a versatile weapon deployed by many bacterial species to deliver diverse effector proteins into eukaryotic host cells or bacterial competitors. The major targets of T6SS antibacterial effectors include the membrane, cell wall, or nucleic acid; some of these are shared by eukaryote-targeting effectors (*e.g.* membranes), whereas the latter may have additional targets (*e.g.* actin cytoskeleton). The delivery and activity of these T6SS toxins and effectors have a clear impact in interbacterial competition and/or pathogenesis during eukaryotic host infection (1, 2). The T6SS apparatus relies on ∼13–14 conserved core components to build a contractile phage tail-like structure anchored to the bacterial cell envelope. To initiate the T6SS assembly, a TssJLM (or TssLM) transmembrane complex (3–6) serves as a docking site for the TssAEFGK baseplate complex (7, 8), with TssK bridging the baseplate and the membrane complex. On the baseplate, Hcp is polymerized in a tail tube-like structure and wrapped around by a TssB-TssC outer sheath. In some studies, it was proposed that TssA is responsible for initiating sheath polymerization (9). Upon contraction of the sheath, the Hcp tube, tipped by the VgrG-PAAR puncturing device, and the T6SS effectors associated with it (10, 11) are propelled across the cell envelope.

T6SS is regulated at multiple levels (12–15). A subset of T6SS gene clusters encode orthologs of serine/threonine kinase PpkA, the cognate phosphatase PppA, and the forkhead-associated (FHA) domain–containing proteins (16), which suggests the involvement of a threonine phosphorylation (TPP) regulatory pathway in these bacteria (17–20). *Pseudomonas aeruginosa* H1-T6SS is post-translationally regulated, positively by PpkA and negatively by the cognate phosphatase PppA. Such control occurs via threonine phosphorylation at the Thr-362 residue on an FHA domain–containing protein, Fha1, in *P. aeruginosa* (21) and *Serratia marcescens* (20). Remarkably, the T6SS inner-membrane protein TssL and not Fha was identified as the substrate of PpkA first in *Agrobacterium tumefaciens* (22) and recently in *Vibiro alginolyticus* (23). In *A. tumefaciens*, TssL forms a stable complex with TssM (4, 6, 24), which exhibits ATPase activity (5). Phosphorylated TssL recruits Fha to the TssM-TssL complex for T6SS activation (22). In *P. aeruginosa*, four type VI secretion–associated genes, namely *tagQRST*, participate in post-translational regulation and act upstream of PpkA to promote kinase activity and subsequent T6SS-dependent secretion (25, 26). Remarkably, the *P. aeruginosa* H1-T6SS can also be activated independently of Fha1 phosphorylation and TPP activity by inactivating a negative regulator, TagF (19), yet the molecular basis underlying the regulatory mechanisms of TagF-mediated T6SS repression has not been addressed.

*A. tumefaciens* harbors one T6SS that is activated at both transcriptional and post-translational levels when sensing acidity (22, 27, 28). Three T6SS effectors, including one peptidoglycan amidase (Tae) and two DNases (Tde1 and Tde2), conferring antibacterial activity, have been identified in this bacterium (29). Autointoxication is prevented through the production of cognate immunity proteins. In *A. tumefaciens*, TagF and PppA are encoded as a fusion protein named TagF-PppA, the overexpression of which abolishes Hcp secretion (22). In this study, we investigated the TagF-PppA suppression mechanism and how this affects T6SS-dependent secretion and antibacterial activity in *A. tumefaciens*. Our data indicate that the TagF domain alone is sufficient to post-translationally repress the T6SS independently of TPP. By performing protein–protein interaction studies, we identified that the cytoplasmic T6SS core component Fha is the binding target for TagF. Using structural modeling, we identified conserved TagF amino acid residues and demonstrated their importance in Fha interaction and TagF-mediated repression of T6SS activity. Remarkably, these conserved residues are also required for *P. aeruginosa* TagF in repressing the H1-T6SS activity and interaction with Fha1. TagF may have evolved while keeping the Fha protein as a target for controlling and preventing T6SS assembly.

## Results

### Both TagF and PppA domains can repress type VI secretion and antibacterial activity at post-translational levels in A. tumefaciens

**Figure 1.**
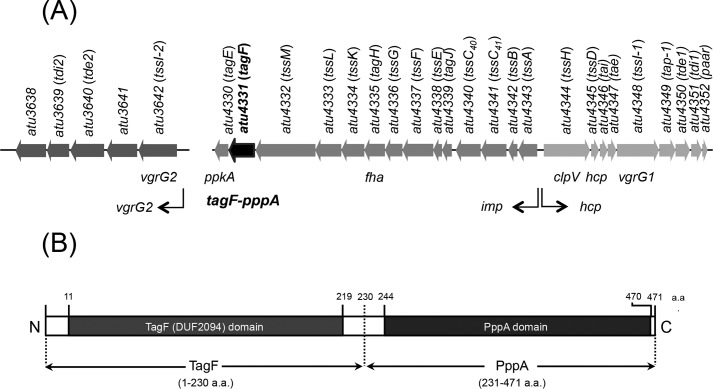
***A. tumefaciens* C58 *t6ss* gene clusters and TagF-pppA domain organization.** *A*, the *imp* operon (*atu4343* to *atu4330*), *hcp* operon (*atu4344* to *atu4352*), and *vgrG2* in *A. tumefaciens* strain C58 were designated *tss* or *tag* based on nomenclature proposed by Shalom *et al.* (34) and specific names derived from Lin *et al.* (30) and Bondage *et al.* (31). *B*, TagF-PppA domain organization according to information from the NCBI conserved domain database. TagF-PppA is predicted as a cytoplasmic protein (aa 1–471) with an N-terminal conserved TagF (DUF2094) domain (aa 11–219) and a C-terminal PppA (PP2Cc) domain (aa 244–470).

**Figure 2.**
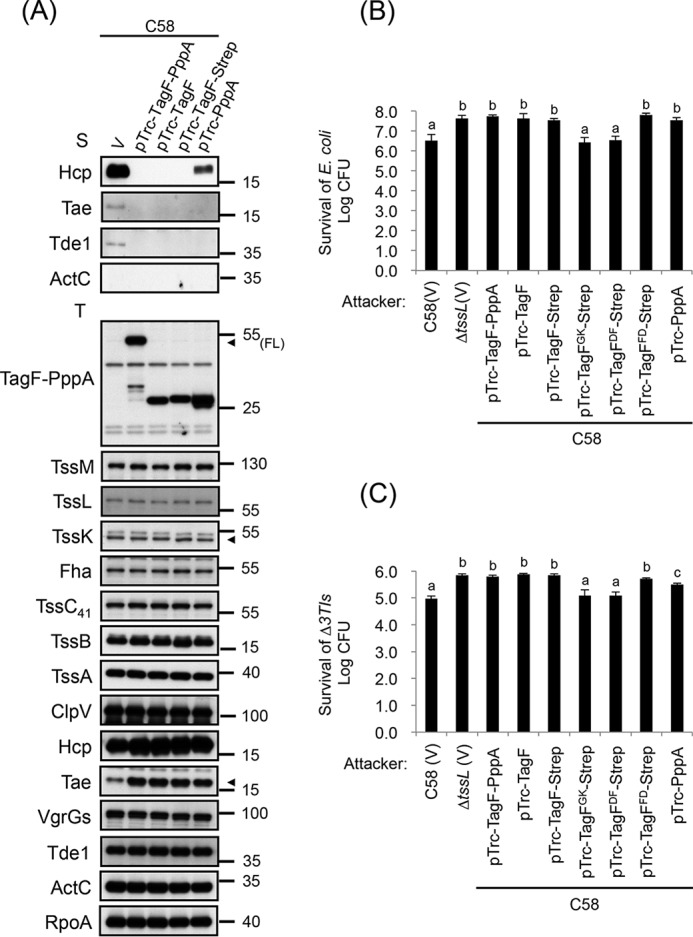
**Both TagF and PppA domains can repress type VI secretion and antibacterial activity at post-translational levels in *A. tumefaciens*.** *A*, type VI secretion analysis. Shown is Western blot analysis of total (*T*) and secreted (*S*) proteins isolated from WT C58 harboring the vector pTrc200 (V) or various overexpressing plasmids grown in AB-MES (pH 5.5) liquid culture with specific antibodies. The nonsecreted protein ActC and RNA polymerase α subunit RpoA were internal controls. The proteins analyzed and sizes of molecular weight standards are shown on the *left* and *right*, respectively, and indicated with *arrowheads* when necessary. *FL*, full-length TagF-PppA protein. *B*, *A. tumefaciens* antibacterial activity assay against *E. coli*. The *A. tumefaciens* WT C58 harboring the vector pTrc200 (V) or various overexpressed plasmids or Δ*tssL* mutant harboring the vector pTrc200 (V) was co-cultured on LB agar with *E. coli* strain DH10B cells harboring the plasmid pRL662. *C*, *A. tumefaciens* intraspecies competition *in planta*. The *A. tumefaciens* WT C58 harboring the vector pTrc200 (V) or various overexpressed plasmids or Δ*tssL* mutant harboring the vector pTrc200 (V) was used as attacker strain to mix with the target strain Δ*3TIs* harboring pRL662 and infiltrated into *N. benthamiana* leaves. *B* and *C*, data are mean ± S.D. of at least three biological replicates. *Different letters above* the *bar* indicate statistically significantly different groups of strains (*p* < 0.01 for *B*; *p* < 0.05 for *C*) based on cfu of the surviving target cells.

TPP regulation of T6SS has been demonstrated in only a few bacteria, including *A. tumefaciens* strain C58 (22). PpkA (Atu4330), TagF-PppA (Atu4331), and Fha (Atu4335), the three major components of the TPP pathway, are all encoded within the *imp* operon (Fig. 1*A*) (22, 27, 30). Atu4331 is a fusion protein, which contains N- and C-terminal domains homologous to TagF (DUF2094) (aa 11–219) and PppA (aa 244–470), respectively (thus named TagF-PppA) (Fig. 1*B*) (22). We previously reported that TagF-PppA plays a negative role in regulating Hcp secretion when TagF-PppA is overexpressed in *A. tumefaciens* (22). However, the molecular mechanism underlying this regulation is unknown. Thus, we first investigated the impact and respective role of individual domain from the TagF-PppA chimera on the TagF-PppA–mediated repression activity on T6SS activity. In *A. tumefaciens* strain C58, we separately produced the TagF domain located at the N terminus (aa 1–230) and the C-terminal region (aa 231–471) encompassing the PppA domain (Fig. 1*B*). Overexpression of TagF-PppA, TagF, and tagged TagF-Strep in C58 completely abolished type VI secretion (Hcp, Tae, and Tde1) (Fig. 2*A*). The T6SS antibacterial activity is also shut down, as shown by counting surviving *Escherichia coli* target cells. The number of survivors is indeed similar to that when the attacker is an *A. tumefaciens* T6SS mutant, Δ*tssL* (Fig. 2*B*). We also performed an *in planta* interbacterial competition assay with an *A. tumefaciens* prey strain lacking the three T6SS toxins (Tae, Tde1, and Tde2) and cognate immunity proteins, Δ*3Tis* (29). Previous observation showed a reduced number of viable prey cells when co-infected with WT C58 (31). Similarly, here, the survival of Δ*3TIs* was lower after co-infection with WT C58 harboring the vector pTrc200 (V). This was not seen when co-infecting with a T6SS inactive mutant Δ*tssL*-carrying pTrc200 (V) or C58 overexpressing the T6SS repressor TagF (*i.e.* TagF-PppA, TagF, or TagF-Strep) (Fig. 2*C*). Interestingly, overexpression of PppA alone reduced but did not abolish Hcp secretion (Fig. 2*A*). The antibacterial activity of the PppA overexpression strain was also not completely abolished, whereas modest antibacterial activity was detected with our *in planta* interbacterial competition assay (Fig. 2*C*). In addition, the protein levels of all analyzed T6SS components encoded within the *imp* (including TssM, TssL, TssK, Fha, TssC_41_, TssB, and TssA) and *hcp* (including ClpV and VgrG1) operons remained the same in all backgrounds tested, including overexpression of TagF-PppA, TagF, TagF-Strep, or PppA (Fig. 2*A*). Taken together, these data show that in *A. tumefaciens*, both TagF and PppA domains contribute the repressor function of TagF-PppA on T6SS effector secretion and antibacterial activity via a post-translational regulatory control.

### Both TagF and PppA domains repress T6SS activity independently of PpkA-mediated TssL phosphorylation

**Figure 3.**
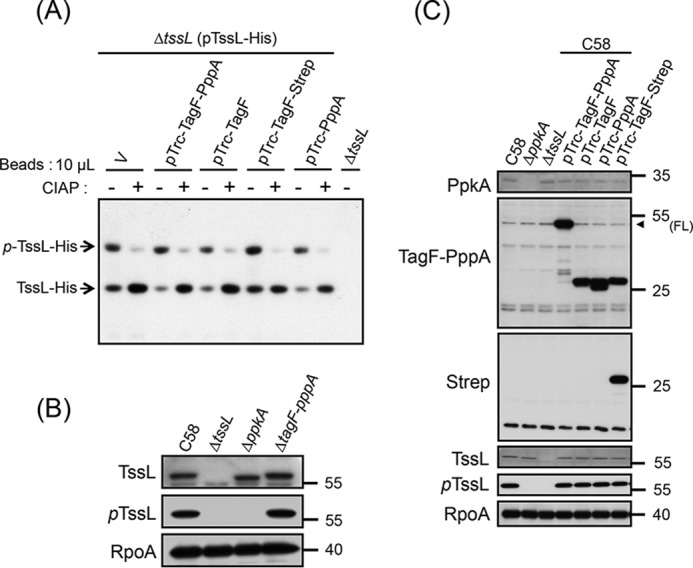
**Both TagF and PppA domains repress T6SS activity independently of the PpkA-mediated TssL phosphorylation pathway in *A. tumefaciens*.** *A*, Phos-tag SDS-PAGE analysis to detect the phosphorylation status of TssL-His. Shown is Western blot analysis of the same volumes of Ni-NTA resins (10 μl) associated with TssL-His from different strains treated with (+) or without (−) CIAP and examined by a specific antibody against His_6_. Total protein isolated from Δ*tssL* was a negative control. Phos-tag SDS-PAGE revealed the upper band indicating the phosphorylated TssL-His (*p-TssL-His*) and lower band indicating unphosphorylated TssL-His. *B* and *C*, Western blot analysis of the endogenous phosphorylation status of TssL (pTssL). Shown is Western blot analysis of total proteins isolated from WT C58, Δ*ppkA*, Δ*tssL*, or C58 harboring the vector pTrc200 (V) or various overexpressing plasmids grown in AB-MES (pH 5.5) liquid culture with specific antibodies. The specific antibody for pTssL was generated against the 15-mer peptide (^7^SSWQDLPpTVVEITEE^21^), with phosphorylated Thr-14 of TssL underlined. RNA polymerase α subunit RpoA was an internal control. The proteins analyzed and molecular weight standards are on the *left* and *right*, respectively, and are indicated with an *arrowhead* when necessary. *FL*, full-length TagF-PppA proteins.

To explore the possible mechanisms of TagF-PppA-mediated T6SS repression at the post-translational level, we analyzed the impact on TssL phosphorylation. TagF-PppA, TagF, TagF-Strep, and PppA were overexpressed in the Δ*tssL* mutant also expressing His-tagged TssL (TssL-His). This TssL variant is functional and mediates Hcp, Tae, and Tde1 secretion (Fig. S1, *A* and *B*) (22). The TssL-His protein was purified by using Ni-NTA resins, and various *A. tumefaciens* strains were analyzed. The Phos-tag SDS-PAGE approach was used, which can detect two TssL-His protein bands, the lower band representing the unphosphorylated TssL-His and the upper band representing the phosphorylated TssL-His (p-TssL-His in Fig. 3*A*) (22). In all cases, the TssL-His protein displayed a similar phosphorylation pattern (Fig. 3*A*), which suggests that neither the full-length TagF-PppA nor any domain of TagF-PppA represses T6SS activity by controlling TssL phosphorylation.

To determine whether the phosphorylation level detected from an overexpressed TssL-His is representative of an endogenous situation, we generated an antibody that specifically recognizes phosphorylated TssL (pTssL) (Fig. 3*B*). Phosphorylated TssL protein was detected as a single protein band and found with the same abundance when comparing WT C58 and strains overexpressing TagF-PppA, TagF, TagF-Strep, and PppA. However, the pTssL protein band was not detected in Δ*tssL* and Δ*ppkA* mutant strains (Fig. 3*C*). We concluded that TagF-PppA represses T6SS activity independently of TssL phosphorylation in *A. tumefaciens*.

**Figure 4.**
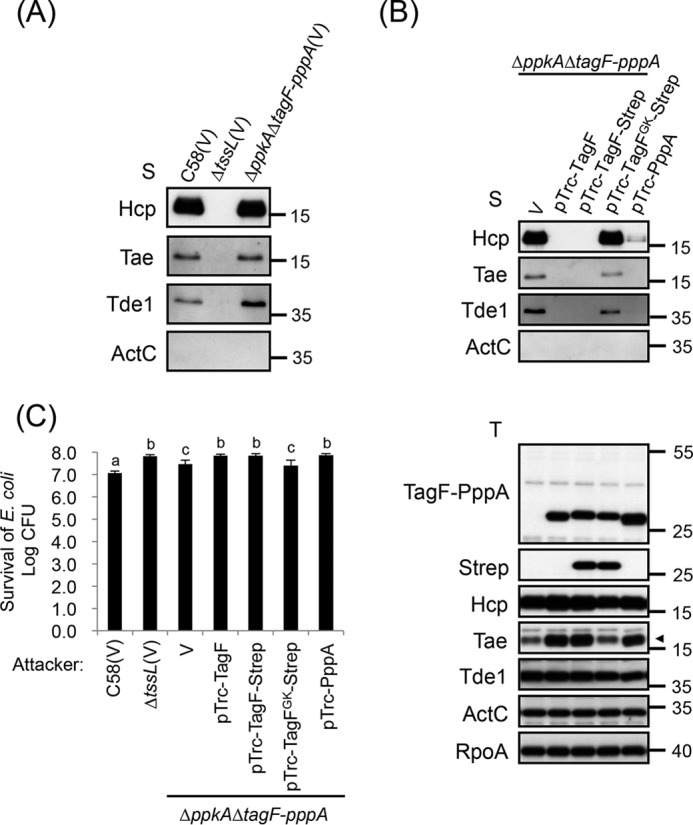
**TagF represses T6SS activity independent of the TPP pathway in *A. tumefaciens*.** *A* and *B*, type VI secretion analysis. Shown is Western blot analysis of total (*T*) and secreted (*S*) proteins isolated from WT C58 harboring the vector pTrc200 (V) or Δ*tssL* harboring the vector pTrc200 (V) or Δ*ppkA*Δ*tagF-pppA* harboring various overexpressing plasmids grown in AB-MES (pH 5.5) liquid culture with specific antibodies. The nonsecreted protein ActC and RNA polymerase α subunit RpoA were internal controls. The proteins analyzed and molecular weight standards are shown on the *left* and *right*, respectively, and indicated with an *arrowhead* when necessary. *C*, *A. tumefaciens* antibacterial activity assay against *E. coli*. The *A. tumefaciens* WT C58 harboring the vector pTrc200 (V), Δ*tssL* harboring the vector pTrc200 (V), or Δ*ppkA*Δ*tagF-pppA* harboring various overexpressing plasmids was co-cultured on LB agar with *E. coli* strain DH10B cells harboring pRL662. Data are mean ± S.D. (*error bars*) of at least three biological replicates. *Different letters above* the *bar* indicate statistically significantly different groups of strains (*p* < 0.01) based on cfu of the surviving target cells.

It is intriguing that overexpression of the PppA domain had no impact on TssL phosphorylation, which is mediated by PpkA, but still could repress T6SS activity. To confirm that the TPP-independent repression of both TagF and PppA domains is not caused by a secondary effect linked to overexpression, we also examined the T6SS secretion and antibacterial activity in a strain lacking the entire TPP (*i.e.* both *ppkA* and *tagF-pppA*). We generated a Δ*ppkA*Δ*tagF-pppA* strain and separately overexpressed the TagF or PppA domain in this mutant background. Interestingly, unlike Δ*ppkA*, resulting in decreased type VI secretion (22), the Δ*ppkA*Δ*tagF-pppA* mutant retained type VI secretion activity comparable with that of the WT C58 (Fig. 4*A*). Overexpression of each of TagF or PppA domain in Δ*ppkA*Δ*tagF-pppA* abolished or greatly reduced the type VI secretion (Fig. 4*B*), suggesting that both TagF and PppA domains can inhibit type VI secretion in the absence of PpkA. As expected, the antibacterial activity was also abolished when the TagF or PppA domain was overexpressed in Δ*ppkA*Δ*tagF-pppA*, as shown by counting *E. coli* survivors and comparison with what is observed when the attacker is a T6SS mutant, Δ*tssL* (Fig. 4*C*). Interestingly, whereas the Hcp and effector (Tde1 and Tae) secretion levels in Δ*ppkA*Δ*tagF-pppA* were comparable with those of WT C58 (Fig. 4*A*), only partial antibacterial activity was observed (Fig. 4*C*). This phenotype is consistent with a previous observation in *P. aeruginosa* and *S. marcescens*, with Δ*pppA* showing reduced antibacterial activity despite elevated type VI secretion (20, 32, 33). These data indicate that both the TagF and PppA domains play a role in repressing the *A. tumefaciens* T6SS activity. This control is exerted at a post-translational level and is independent of PpkA and TssL phosphorylation.

### TagF interacts with the forkhead-associated protein Fha of A. tumefaciens and P. aeruginosa

**Figure 5.**
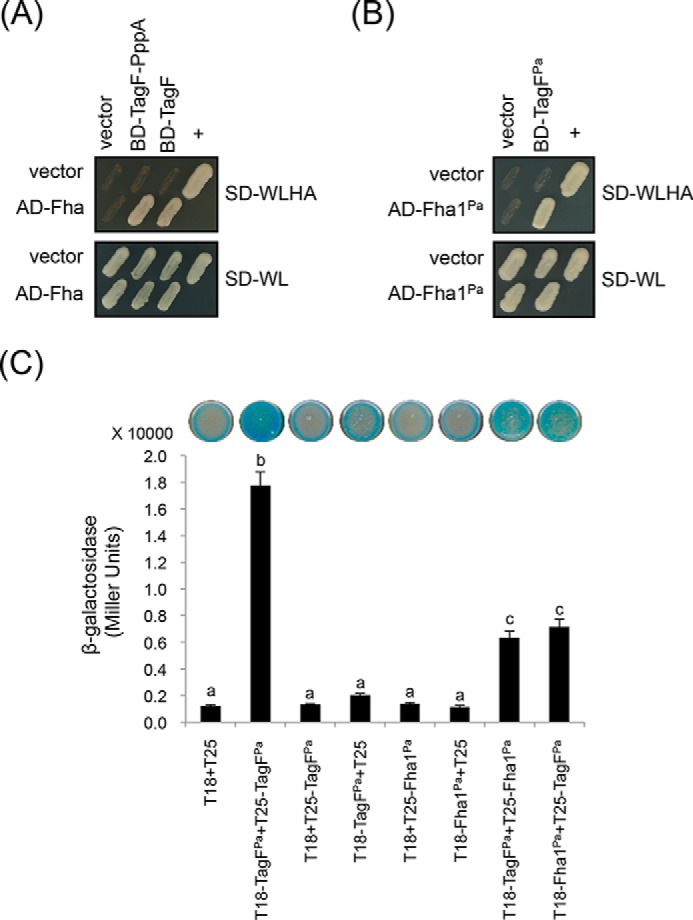
**TagF directly interacts with Fha of *A. tumefaciens* and *P. aeruginosa*.** *A* and *B*, yeast two-hybrid protein–protein interaction results. SD−WL medium (SD minimal medium lacking Trp and Leu) was used for the selection of plasmids. SD−WLHA medium (SD minimal medium lacking Trp, Leu, His, and Ade) was used for the auxotrophic selection of bait and prey protein interactions. The positive interaction was determined by growth on SD−WLHA medium at 30 °C for at least 2 days. The positive control (+) showing interactions of SV40 large T-antigen and murine p53 and negative control (vector) are indicated. *C*, bacterial two-hybrid analysis. Various combinations of recombinant pKT25 and pUT18C plasmids harboring *P. aeruginosa* TagF^Pa^ or Fha1^Pa^ proteins were co-transformed into *E. coli*. A graphical representation of the β-gal activity from co-transformants is shown, the plasmid combinations are indicated *below*, and images of corresponding *E. coli* spots on LB agar plates containing X-gal are displayed at the *top*. The strength of the interaction was investigated by measuring the β-gal activity of cells. The average activity in Miller units is indicated. Experiments were carried out in duplicate, and data are mean ± S.D. (*error bars*) *Different letters above* the *bar* indicate statistically significantly different groups (*p* < 0.01). *T18*, empty vector pUT18C; *T25*, empty vector pKT25.

Next, we investigated how TagF-PppA represses T6SS activity via a post-translational and TPP-independent pathway. We hypothesized that TagF-PppA may interact with the T6SS core component(s) to prevent T6SS activation. One plausible candidate is the forkhead-associated protein Fha because of its role in recruitment to a membrane-associated complex both independent of and dependent on TPP (19, 21, 22). Because TagF is the common repressor known to suppress T6SS independently of TPP in both *A. tumefaciens* and *P. aeruginosa*, we set up experiments to determine whether *A. tumefaciens* TagF-PppA and *P. aeruginosa* TagF can interact with their cognate Fha. Yeast two-hybrid (YTH) experiments revealed that both *A. tumefaciens* full-length TagF-PppA and the isolated TagF domain specifically interact with Fha (Fig. 5*A*). We also detected an interaction between the *P. aeruginosa* H1-T6SS–encoded TagF and Fha1 by using both YTH (Fig. 5*B*) and bacterial two-hybrid (BTH) assays (Fig. 5*C*). The data demonstrate that TagF directly interacts with Fha, probably interfering with its function and thereby preventing type VI secretion in both *A. tumefaciens* and *P. aeruginosa*.

### Conserved amino acid residues in TagF are critical for TagF–Fha interaction

**Figure 6.**
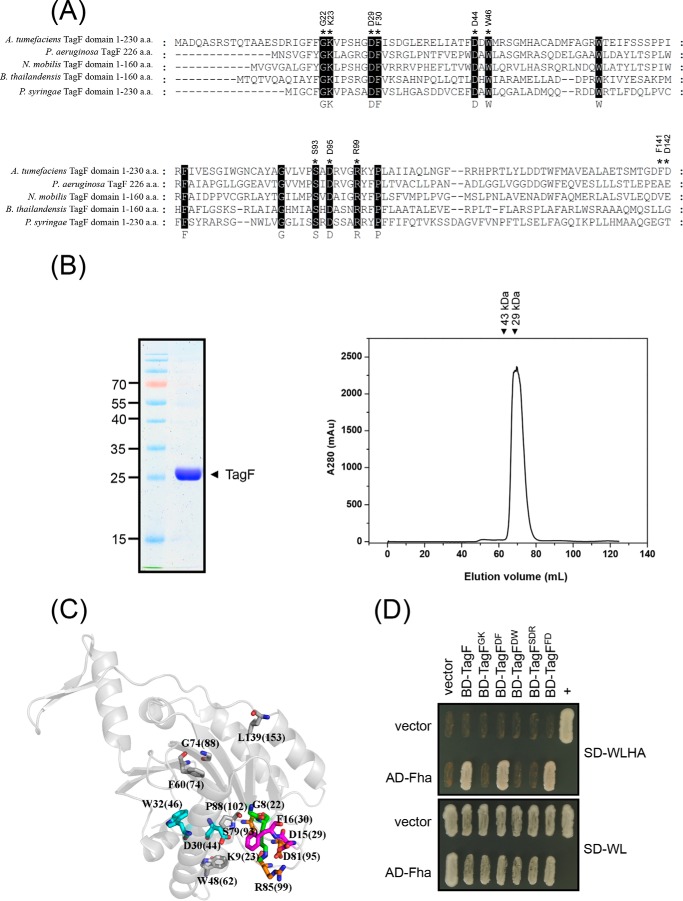
**Conserved amino acid residues of TagF are critical for TagF–Fha interaction in *A. tumefaciens*.** *A*, amino acid sequence alignment of TagF or TagF domain orthologs from selected bacterial species. Conserved amino acid residues are *highlighted* in *black*, and those used for mutagenesis are indicated with an *asterisk*. Sequences were aligned and highlighted by use of ClustalW2 (http://www.ebi.ac.uk/Tools/msa/clustalw2/). (Please note that the JBC is not responsible for the long-term archiving and maintenance of this site or any other third party hosted site.) Part of the aligned result is shown here, and the fully aligned result and full information for bacterial strains and protein accession numbers are shown in Fig. S3*A*. *B*, *Agrobacterium* TagF protein is present as a monomer on gel filtration analysis *in vitro*. Purified His-tagged TagF domain (aa 1–214) was analyzed by SDS-PAGE. The proteins analyzed and molecular weight standards are shown on the *right* and *left*, respectively. His-tagged TagF proteins were further analyzed by use of a Superdex 75 16 × 60 column, and the elution profiles were recorded as absorbance at 280 nm showing that His-tagged TagF elutes as a single peak (∼26 kDa monomer). *C*, relative positions of the conserved amino acid residues in *P. aeruginosa* TagF^Pa^ protein revealed as a monomer with crystal structural information according to the X-ray crystal structure of *P. aeruginosa* TagF monomer (Protein Data Bank entry 2QNU). The corresponding conserved amino acid residues of *A. tumefaciens* TagF are indicated in *parenthesis. D*, yeast two-hybrid protein–protein interaction results with Fha and various TagF proteins. SD−WL medium (SD minimal medium lacking Trp and Leu) was used for selecting plasmids. SD−WLHA medium (SD minimal medium lacking Trp, Leu, His, and Ade) was used for the auxotrophic selection of bait and prey protein interactions. The positive interaction was determined by growth on SD−WLHA medium at 30 °C for at least 2 days. The positive control (+) showing interactions of SV40 large T-antigen and murine p53 and negative control (vector) are indicated.

Although Fha is the target for TagF in both *A. tumefaciens* and *P. aeruginosa*, the two TagF proteins share only limited amino acid similarity (Fig. 6*A*). Yet, 14 amino acid residues are highly conserved among various TagF orthologs (Fig. 6*A* and Fig. S3*A*). We hypothesized that these residues may play important roles in TagF function. The structure of *P. aeruginosa* TagF is presented as a homodimer (Protein Data Bank entry 2QNU). The self-interaction of *P. aeruginosa* TagF was also confirmed by BTH and YTH (Fig. 5*C* and Fig. S2*A*). In contrast, the *A. tumefaciens* TagF probably does function as a monomer, as supported by several lines of evidence. First, the TagF protein is mostly a monomer when analyzed by gel filtration *in vitro* (Fig. 6*B*). Second, *A. tumefaciens* TagF lacks the residues required for dimer formation in *P. aeruginosa* (Val-105 (*V105*), Leu-169 (*L169*), Leu-172 (*L172*), Ala-173 (*A173*), and Leu-195 (*L195*) in TagF^Pa^) (Fig. S3*A*) (Protein Data Bank entry 2QNU). Finally, no self-interaction could be found for *A. tumefaciens* TagF using YTH (Fig. S2*B*). Other conserved residues are not involved in *P. aeruginosa* TagF homodimerization and were thus chosen for site-directed mutagenesis and characterization of the potential interface with other interacting proteins, namely Fha. A total of eight conserved residues (Gly-8 (*G8*), Lys-9 (*K9*), Asp-15 (*D15*), Phe-16 (*F16*), Ser-79 (*S79*), Asp-81 (*D81*), Arg-85 (*R85*), and Pro (*P88*) of *P. aeruginosa* TagF) form a specific loop, and the side chain of Asp-30 (*D30*) is outwardly exposed (Fig. 6*C*). Therefore, these are potential sites to interact with other proteins. In contrast, Phe-60 (*F60*), Gly-74 (*G74*), and Leu-139 (*L139*) are located inside the structure or the side chain wrapped in an internal structure to prevent interaction with other proteins. We generated four mutants with amino acid substitutions of alanine at GK (G8K9 in *P. aeruginosa* TagF; G22K23 in *A. tumefaciens* TagF), DF (D15F16 in *P. aeruginosa* TagF; D29F30 in *A. tumefaciens* TagF), DW (D30W32 in *P. aeruginosa* TagF; D44W46 in *A. tumefaciens* TagF), and SDR (S79D81R85 in *P. aeruginosa* TagF; S93D95R99 in *A. tumefaciens* TagF) (Fig. 6, *A* and *C*). We performed YTH analysis with the various *A. tumefaciens* TagF variants to determine the roles of the substituted residues in the TagF–Fha interaction. The interaction was completely lost in TagF^GK^, TagF^DW^, and TagF^SDR^ mutants (Fig. 6*D*). In contrast, TagF^DF^ and TagF^FD^ with mutations in two randomly selected nonconserved residues (Phe-141 and Asp-142 (F141D142) in *A. tumefaciens* TagF) still retained full capacity to interact with Fha as compared with the WT TagF (Fig. 6*D*). Western blot analysis revealed that the protein abundance remained the same or was even slightly higher for all analyzed TagF variants as compared with the WT TagF (Fig. S3*B*), which indicates that conserved G^22^K^23^, D^44^W^46^, and S^93^D^95^R^99^ residues but not D^29^F^30^ of *A. tumefaciens* TagF are critical for the TagF–Fha interaction.

### Loss of TagF–Fha interaction upon site-directed mutagenesis abolishes the repression of T6SS activity in A. tumefaciens

**Figure 7.**
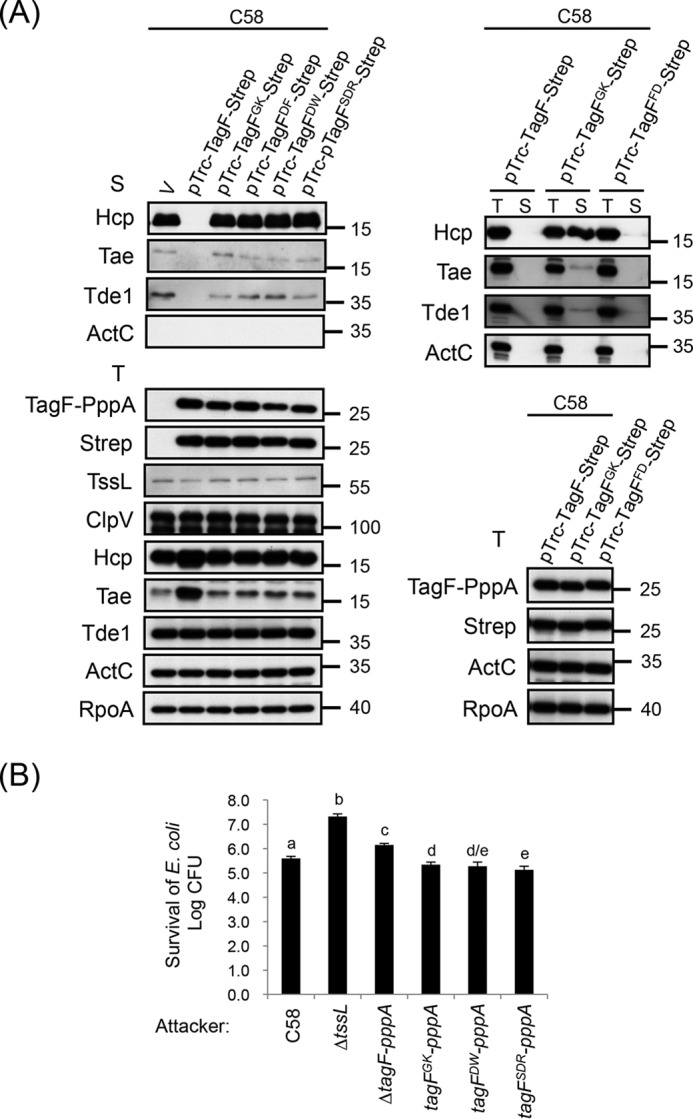
**Conserved amino acid residues of TagF are required for repressing type VI activity in *A. tumefaciens*.** *A*, Western blot analysis of total (*T*) and secreted (*S*) proteins isolated from WT C58 harboring the vector pTrc200 (V) or various TagF-Strep–overexpressing plasmids grown in AB-MES (pH 5.5) liquid culture with specific antibodies. The nonsecreted protein ActC and RNA polymerase α subunit RpoA were internal controls. The proteins analyzed and molecular weight standards are shown on the *left* and *right*, respectively. *B*, *A. tumefaciens* antibacterial activity assay against *E. coli*. The *A. tumefaciens* WT C58 or Δ*tssL* or chromosomally encoded *tagF-pppA* variants, including *tagF-pppA* with substitutions in the *tagF* domain (*tagF^GK^-pppA*, *tagF^DW^-pppA*, and *tagF^SDR^-pppA*), were co-cultured on LB agar with *E. coli* strain DH10B cells harboring the plasmid pRL662. Data are mean ± S.D. (*error bars*) of at least three biological replicates. *Different letters above* the *bar* indicate statistically significantly different groups of strains (*p* < 0.05) based on cfu of the surviving target cells.

To determine whether the TagF–Fha interaction is required for suppressing T6SS activity, we engineered the previously described mutations in the TagF-Strep variants and analyzed the impact of their overexpression on type VI secretion. As expected, overexpression of the WT controls, TagF-Strep or TagF^FD^-Strep, abolished Hcp, Tae, and Tde1 secretion. Remarkably, the secretion capacity remained high with overexpression of TagF^GK^-Strep, TagF^DF^-Strep, TagF^DW^-Strep, and TagF^SDR^-Strep in C58 (Fig. 7*A*). Upon Western blot analysis, the protein levels of all TagF variants were comparable with that of WT TagF-Strep, and overexpression did not affect the protein abundance of other T6SS components (Fig. 7*A*). These data suggest that the conserved residues GK, DW, and SDR of TagF are critical for TagF-mediated repression of type VI secretion via the TagF–Fha interaction. Intriguingly, TagF^DF^ retained full binding capacity with Fha but lost the ability to repress type VI secretion, which suggests that D^29^F^30^ is not involved in binding Fha but is required for repressing T6SS activity. As expected, the type VI secretion activity of these TagF overexpression variants was consistent with their antibacterial activity (Fig. 2, *B* and *C*). The survival of *E. coli* was reduced to a level similar to that with the strain harboring the empty vector control when TagF^GK^-Strep was overexpressed in Δ*ppkA*Δ*tagF-pppA* (Fig. 4, *B* and *C*), which confirms that TagF^GK^-Strep lost the ability to repress T6SS-dependent antibacterial activity via a PpkA-independent pathway. To ensure that the overexpression phenotypes of the mutant alleles were also reflected when expressed at endogenous levels, we further generated chromosomal *tagF-pppA* alleles encoding the TagF amino acid substitution variants and further determined their T6SS suppression activity. All TagF amino acid substitution variants expressed from chromosomal alleles exhibited enhanced antibacterial activity (Fig. 7*B*), which is consistent with the derepressing T6SS activity demonstrated upon overexpression of these TagF variants (Fig. 2, *B* and *C*). However, our secretion assay could not detect a significant difference in secretion levels of Hcp and two effectors (Tae and Tde1) of all analyzed *tagF-pppA* mutants as compared with WT C58 (Fig. S4). Taken together, our data suggest that TagF negatively regulates T6SS via direct interaction with Fha in *A. tumefaciens*.

### Overexpression of TagF causes reduced T6SS protein accumulation and abolishes T6SS antibacterial activity in P. aeruginosa

**Figure 8.**
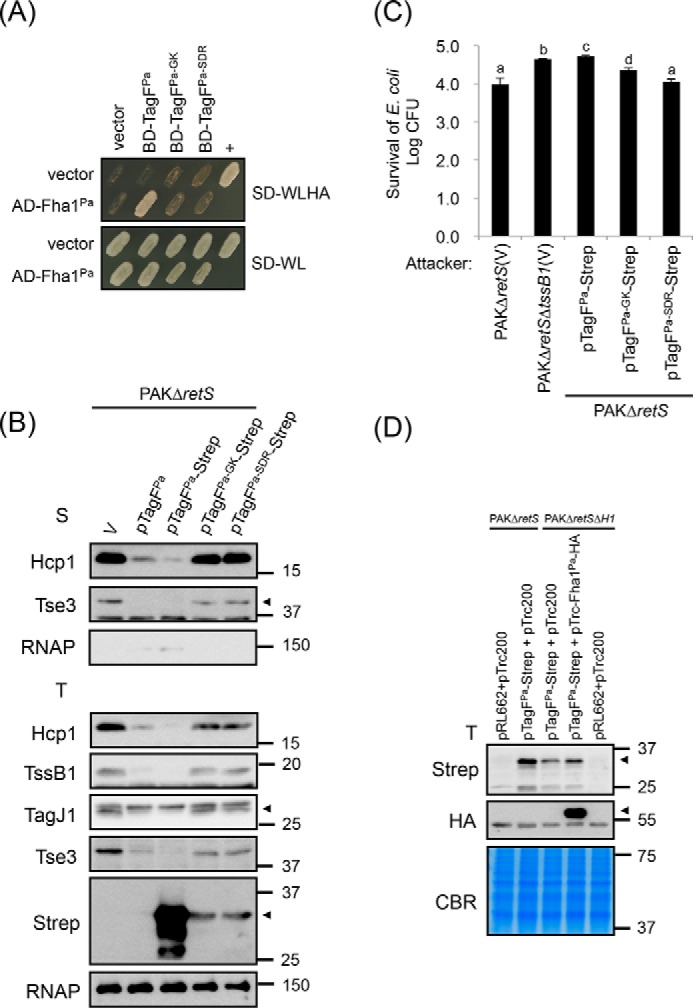
**Conserved amino acid residues of TagF^Pa^ critical for TagF^Pa^–Fha1^Pa^ interaction are required for repressing H1-T6SS activity in *P. aeruginosa*.** *A*, yeast two-hybrid protein–protein interaction results with *P. aeruginosa* Fha1 and various *P. aeruginosa* TagF proteins. SD−WL medium (SD minimal medium lacking Trp and Leu) was used for selecting plasmids. SD−WLHA medium (SD minimal medium lacking Trp, Leu, His, and Ade) was used for auxotrophic selection of bait and prey protein interactions. The positive interaction was determined by growth on SD−WLHA medium at 30 °C for at least 2 days. The positive control (+) showing interactions of SV40 large T-antigen and murine p53 and negative control (vector) are indicated. *B*, *P. aeruginosa* H1-T6SS secretion analysis. Shown is Western blot analysis of total (*T*) or secreted (*S*) proteins isolated from *P. aeruginosa* PAKΔ*retS* (H1-T6SS–induced) harboring the vector pRL662 (V) or PAKΔ*retS* harboring various overexpressed plasmids grown in TSB with specific antibodies. The nonsecreted RNA polymerase β subunit (*RNAP*) was an internal control. The proteins analyzed and molecular weight standards are shown on the *left* and *right*, respectively, and indicated with an *arrowhead* when necessary. *C*, *P. aeruginosa* H1-T6SS–mediated antibacterial assay against *E. coli*. Overnight cultures of *P. aeruginosa* PAKΔ*retS* or PAKΔ*retS*Δ*tssB1* (T6SS-defective strain) harboring the vector pRL662 (V) or various *tagF-Strep*–overexpressing plasmids were mixed with equivalent numbers of *E. coli* DH5α carrying a plasmid (pCR2.1) expressing β-gal. Data are mean ± S.D. of at least three biological replicates. *Different letters above* the *bar* indicate statistically significantly different groups of strains (*p* < 0.05) based on cfu of the surviving target cells. *D*, the presence of Fha1^Pa^ increases the stability of TagF^Pa^ protein in *P. aeruginosa*. Shown is Western blot analysis of total (*T*) proteins isolated from *P. aeruginosa* PAKΔ*retS* (H1-T6SS–induced) or PAKΔ*retS*Δ*H1* (deletion of *retS* and *H1-T6SS* cluster) harboring various plasmid combinations grown in TSB with specific antibodies. All protein samples were analyzed by SDS-PAGE followed by Coomassie Blue staining (*CBR*) and served as an internal control. The proteins analyzed and molecular weight standards are shown on the *left* and *right*, respectively, and indicated with *arrowheads* when necessary.

We then assessed whether these specific conserved amino acid residues of TagF required for binding to Fha and T6SS repression in *A. tumefaciens* are also required in *P. aeruginosa* TagF for Fha1^Pa^ interaction and T6SS activity. We generated two alanine substitution mutants in TagF^Pa^, namely TagF^Pa-GK^ (Gly-8 and Lys-9) and TagF^Pa-SDR^ (Ser-79, Asp-81, and Arg-85). Consistent with the results obtained with *A. tumefaciens*, TagF^Pa-GK^ and TagF^Pa-SDR^ mutants lost the interaction with Fha1^Pa^, although their expression level was comparable with that of WT TagF^Pa^ in yeast (Fig. 8*A* and Fig. S3*C*). To determine whether the TagF^Pa^–Fha1^Pa^ interaction is critical for TagF-dependent H1-T6SS repression, WT TagF^Pa^, Strep-tagged WT TagF^Pa^, and the TagF^Pa-GK^ and TagF^Pa-SDR^ variants were expressed from pRL662 in the *P. aeruginosa* Δ*retS* mutant, a constitutively H1-T6SS active strain (35). As expected, Hcp1 and Tse3 were secreted into the culture medium of Δ*retS* harboring the vector pRL662 (V), but H1-T6SS secretion was greatly reduced when TagF^Pa^ or TagF^Pa^-Strep was overexpressed (Fig. 8*B*). Furthermore, overexpression of TagF^Pa-GK^-Strep or TagF^Pa-SDR^-Strep did not repress Hcp1 and Tse3 secretion. Interestingly, in contrast to *A. tumefaciens*, where overexpressed TagF mutant variants are at a level comparable with the overexpressed WT form, in *P. aeruginosa*, we observed significantly higher levels of WT TagF^Pa^-Strep as compared with TagF^Pa-GK^-Strep or TagF^Pa-SDR^-Strep (Fig. 8*B*). Furthermore, protein levels of all analyzed T6SS components (including secreted proteins Hcp1 and Tse3 and structural proteins TssB1 and TagJ1) were significantly reduced upon overexpression of WT TagF (TagF^Pa^ or TagF^Pa^-Strep). Accordingly, protein levels of these T6SS components were restored to near WT levels in the presence of TagF^Pa-GK^-Strep and TagF^Pa-SDR^-Strep in *P. aeruginosa* (Fig. 8*B*).

Next, we performed an antibacterial activity assay and showed that when TagF^Pa^-Strep was overexpressed in PAKΔ*retS*, *E. coli* survival is similar to that for the T6SS-defective PAKΔ*retS*Δ*tssB1* mutant. In contrast, the expression of TagF^Pa-GK^-Strep and TagF^Pa-SDR^-Strep conferred antibacterial activity similar to that of PAKΔ*retS* (Fig. 8*C*). Because the reduced protein level/stability of both TagF^Pa-GK^-Strep and TagF^Pa-SDR^-Strep is associated with loss of ability to interact with Fha1^Pa^, we tested whether the presence or absence of Fha1^Pa^ affected TagF abundance. Strikingly, TagF^Pa^-Strep protein level was significantly reduced in PAKΔ*retS*Δ*H1*, which lacks endogenous Fha1^Pa^, as compared with PAKΔ*retS* (Fig. 8*D*). TagF^Pa^-Strep protein levels could be partially restored upon co-expression of TagF^Pa^-Strep and Fha1^Pa^-HA in PAKΔ*retS*Δ*H1* (Fig. 8*D*), which suggests that Fha1^Pa^ may play a role in stabilizing TagF^Pa^ in *P. aeruginosa*. Taken together, our results indicate that the TagF domain represses T6SS activity via interaction with Fha but with distinct mechanisms when comparing *A. tumefaciens* and *P. aeruginosa.*

## Discussion

In the present study, we have characterized the *A. tumefaciens* TagF-PppA and *P. aeruginosa* TagF and provide compelling evidence that TagF specifically interacts with Fha to repress type VI secretion and antibacterial activity independently of TPP. The loss of interaction with Fha is associated with loss of repression of T6SS activity in both cases, yet we observed differences in the nature and impact of TagF and its variants in repressing T6SS. This may have strategic consequences for how bacteria from various species communicate and respond to each other during T6SS-dependent bacterial warfare.

One remarkable difference is that TagF–Fha interaction has a different impact on T6SS protein abundance between *A. tumefaciens* and *P. aeruginosa*. A similar protein abundance of all TagF^At^ or TagF^At^-Strep overexpressed (WT and all mutants) was observed in *A. tumefaciens.* In contrast, in *P. aeruginosa*, the protein level was significantly lower for TagF^Pa-GK^-Strep or TagF^Pa-SDR^-Strep than WT TagF^Pa^-Strep (Figs. 2*A*, 7*A*, and 8*B*). Furthermore, all analyzed T6SS proteins accumulated to a similar level in the presence or absence of endogenous or overexpressed TagF^At^ in *A. tumefaciens* (Figs. 2*A* and 7*A*). However, the protein levels of *P. aeruginosa* H1-T6SS components (including secreted proteins Hcp1 and Tse3 and structural proteins TssB1 and TagJ1) were reduced with TagF^Pa^ or TagF^Pa^-Strep overexpression but restored to nearly WT levels in the presence of mutant TagF^Pa-GK^-Strep or TagF^Pa-SDR^-Strep (Fig. 8*B*). Previous studies in *P. aeruginosa* suggested that TagF^Pa^-mediated T6SS repression is mediated via a post-translational regulation mechanism (19). The evidence is based on the lack of influence on the expression of *lacZ*-translational fusion to *fha1* or *tssA1* and on the levels of two secreted proteins, Hcp and Tse1, in a Δ*tagF* mutant, which activates Hcp and Tse1 secretion. Instead of analyzing a *tagF* mutant here, we used strains overexpressing TagF, and our data suggest that the *P. aeruginosa* TagF-mediated post-translational repression occurs via influencing the protein stability of T6SS components when TagF^Pa^ is expressed in excess amounts. Taken together, Fha is a common target for TagF in repressing T6SS via post-translational regulation in both *A. tumefaciens* and *P. aeruginosa*, but each has a different strategy to exert this repression activity.

The role of Fha in activating T6SS in both TPP-dependent and -independent pathways may provide some clues to understand the mode of action by which TagF represses T6SS activity. Fha1 but not its phosphorylated form is required for ClpV1^Pa^ foci formation and TagF^Pa^-mediated derepression of type VI secretion in *P. aeruginosa* (19, 21). Also, Fha protein is a core component for T6SS in several bacteria lacking PpkA, PppA, and TagF (21, 22, 30, 36). Therefore, in addition to functioning as a scaffold protein specifically binding a phosphothreonine protein, Fha may also serve as a core T6SS component, probably via interaction with one or multiple T6SS components to activate T6SS assembly and secretion. TssM could be such a candidate because Fha1 foci formation is lost in a *P. aeruginosa* Δ*icmF(tssM*) mutant (37). In *A. tumefaciens*, Fha specifically interacts with TssL at phosphothreonine 14 to associate with the TssM-TssL inner membrane complex and recruit Hcp to interact with TssL for activating type VI secretion (4, 22). Together with our current knowledge of the T6SS assembly pathway (2, 10, 38), these studies indicate that Fha plays a key role in T6SS assembly at step(s) before recruitment of Hcp and TssB-TssC for tail polymerization. In *A. tumefaciens*, only WT TssL but not a TssL variant with the T14A amino acid substitution could interact with Fha, as assessed by a pulldown assay (22). Thus, Fha may interact with another core component(s) of the T6SS in addition to TssL or Fha itself, especially when PpkA is absent or not active. If so, TagF may compete with other T6SS core components for interacting with Fha and thereby prevent Fha from binding to a T6SS membrane–associated complex for stability (in *P. aeruginosa*) or activation of T6SS assembly (in *A. tumefaciens*).

Of note, the conserved G^22^K^23^, D^44^W^46^, and S^93^D^95^R^99^ residues but not D^29^F^30^ of *A. tumefaciens* TagF are critical for the TagF–Fha interaction, but all are required for repressing T6SS activity. This result led us to propose that D^29^F^30^ may repress T6SS function independently of binding to Fha. TagF may target Fha in repressing T6SS activity in two steps. The first step is to bind Fha via an interface involving G^22^K^23^, D^44^W^46^, and S^93^D^95^R^99^. Upon binding, TagF interferes with Fha recruitment to the membrane-associated complex for T6SS activation via D^29^F^30^ residues. Alternatively, it is possible that TagF can also target other T6SS components in addition to Fha. Future work to elucidate the molecular details underlying how TagF–Fha interaction influences T6SS activity or identifying additional TagF-interacting partners may provide answers to distinguish between two possible mechanisms.

**Figure 9.**
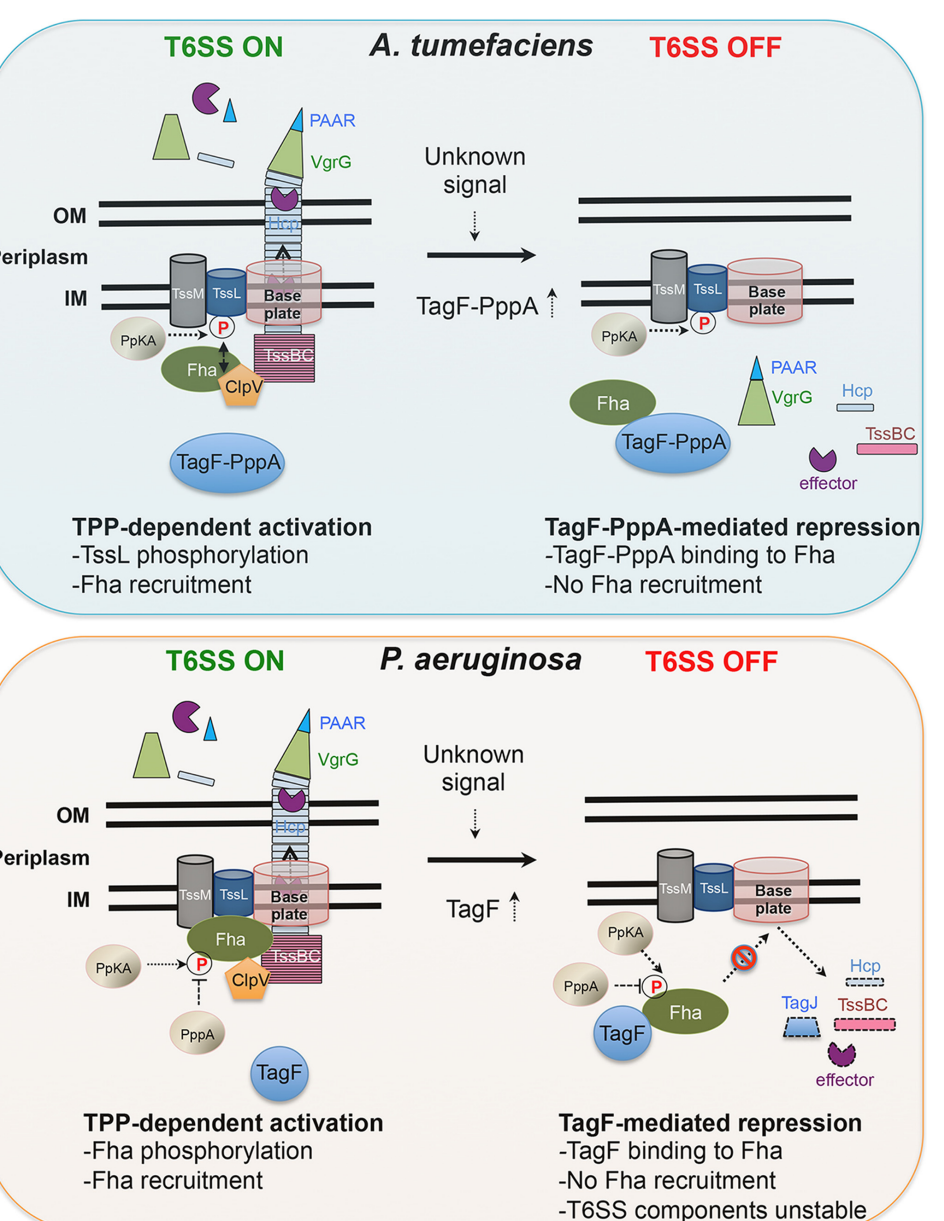
**Proposed models of TPP activation and TagF-mediated post-translational repression of type VI secretion in *A. tumefaciens* and *P. aeruginosa*.** Proposed models of TPP activation (*T6SS ON*) and TagF-mediated repression (*T6SS OFF*) in *A. tumefaciens* (*top*) and *P. aeruginosa* (*bottom*) are illustrated. Key activation or repression events are summarized at the *bottom* of each model. Protein names are indicated *in* or *near* the designated molecules. *IM*, inner membrane; *OM*, outer membrane. For a detailed description of the proposed models, see “Discussion.”

Combining previous (19, 21, 22) and current findings, we propose distinct models for TagF-mediated T6SS repression in *A. tumefaciens* and *P. aeruginosa* (Fig. 9). In WT *A. tumefaciens* when PpkA is active, the level of endogenous TagF-PppA is very low, and the protein does not bind to Fha, with no or little repression activity observed because Fha would then bind to pTssL for triggering T6SS assembly and secretion (T6SS ON shown in the *top left panel* of Fig. 9). On sensing an unknown signal, which may cause high accumulation of TagF-PppA or suppression of the TPP pathway, TagF-PppA can interact with Fha via its TagF domain to prevent it from binding to the membrane-associated complex and thus preventing T6SS activation (T6SS OFF shown in the *top right panel* of Fig. 9). Because TssM and TssL can form an inner-membrane complex in the absence of TssL phosphorylation (4, 22), the TssM-TssL complex and the associated baseplate complex probably remain properly assembled in the membrane when T6SS is suppressed by TagF-PppA. However, Hcp and TssBC may not be polymerized into the tail-like structure, and effector proteins are not loaded on the VgrG-PAAR spike for secretion. Because type VI secretion can be restored to the WT level in the Δ*ppkA*Δ*tagF-pppA* mutant (Fig. 4*A*), Fha probably also functions as a core T6SS component via interaction with one or multiple T6SS components to activate T6SS assembly and secretion in the absence of PpkA and TagF-PppA. This proposed mechanism also explains the previous observation that type VI secretion is highly attenuated but not completely abolished in the absence of PpkA (Δ*ppkA*) (22, 30), because endogenous TagF-PppA, albeit at a low level, can bind Fha, and only a fraction of the Fha pool is available for recruitment to the T6SS core complex.

In *P. aeruginosa*, type VI secretion is significantly enhanced in Δ*pppA* or Δ*tagF* as compared with the parental strain, and PppA phosphatase negatively regulates type VI secretion in a TPP-dependent manner, whereas TagF represses type VI secretion independently of TPP (19, 21). Thus, in the WT *P. aeruginosa* strain harboring both TPP components and TagF, type VI secretion remains at low levels, probably because of a series of phosphorylation and dephosphorylation events as well as TagF interaction with a fraction of the Fha1 pool (T6SS ON shown in the *bottom left panel* of Fig. 9). When TagF is expressed in excess amounts, TagF interacts with Fha to prevent it from binding to T6SS components and thus from activating T6SS assembly and subsequent secretion. Failure to recruit Fha1 to the membrane-associated T6SS complex may send out a signal to trigger degradation of cytoplasmic T6SS components and effectors (T6SS OFF shown in the *bottom right panel* of Fig. 9). Our current study may also provide an explanation for the enhanced T6SS secretion and antibacterial activity in a Δ*tagF* mutant in the presence or absence of PpkA (19, 21). Because Fha1 protein itself but not its phosphorylation is required for ClpV1^Pa^ foci formation (19, 21), nonphosphorylated Fha1 may remain active in binding membrane-associated T6SS component(s), thus resulting in T6SS assembly and secretion in *P. aeruginosa*. With no TagF functionally available (*i.e.* in the absence of the protein or presence of a TagF mutant losing Fha1-binding activity), all Fha1 is available for activating T6SS assembly and secretion.

In conclusion, our proposed molecular model may provide answers to the longstanding question of how TagF mediates T6SS repression. We present compelling evidence suggesting that TagF specifically interacts with Fha and that such binding prevents Fha from being recruited to a T6SS membrane-associated complex. The TagF–Fha interaction has a different impact in different bacteria but ultimately prevents successful T6SS assembly. This study adds to our understanding of how bacteria deploy TPP-dependent activation and TPP-independent TagF-mediated repression mechanisms to control T6SS.

## Experimental procedures

### Bacterial strains, plasmids, and growth conditions

Strains, plasmids, and primer sequences used in this study are described in Tables S1 and S2. The growth conditions are described in detail in supporting information S1.

### Plasmid construction and generation of in-frame deletion mutants

All in-frame deletion mutants were generated in *A. tumefaciens* C58 via double crossover using the suicide plasmid pJQ200KS (39) as described previously (4, 27). The detailed procedures for the construction of plasmids and mutant strains are described in supporting information S1.

### Antibody production

The specific antibody for phosphorylated TssL (pTssL) was generated against the 15-mer peptide (^7^SSWQDLPpTVVEITEE^21^) containing the phosphorylated Thr-14 residue (22). The pTssL epitope located at the N-terminal region of TssL was used for polyclonal antibody production in rabbits.

### A. tumefaciens type VI secretion and antibacterial competition assays

Type VI secretion assay was performed as described previously (4, 27, 29, 30). To study type VI secretion from *A. tumefaciens* grown in liquid medium, *A. tumefaciens* cells were grown in liquid 523 medium for 16 h at 25 °C. Cells were harvested, and *A*_600_ was adjusted to 0.1. *A. tumefaciens* cells continued to grow in liquid AB-MES medium (pH 5.5) (40) at 25 °C for 6 h. The cell suspension was centrifuged at 10,000 × *g* for 15 min at 4 °C; the resulting supernatant was concentrated by TCA precipitation, and Hcp, Tae, and Tde1 secretion was monitored as described previously (4, 27). An *A. tumefaciens* antibacterial activity assay using *E. coli* as target cells was performed as described previously (29). In brief, overnight grown *A. tumefaciens* and *E. coli* strains harboring appropriate plasmids were adjusted to *A*_600_ of 0.1 and incubated at 25 °C for 4–5 h before co-incubation. *A. tumefaciens* and *E. coli* cells were harvested, and *A*_600_ was adjusted, and then cells were mixed at a 1:30 ratio (*A*_600_ 0.01:0.3) and spotted onto LB agar plates. Where applicable, the mixture was spotted onto an LB agar plate containing 0.5 mm isopropyl β-d-thiogalactopyranoside (IPTG) to induce expression from the pTrc200 plasmid. 16 h after incubation at 25 °C, the spots were harvested, serially diluted, and plated on LB agar plate containing appropriate antibiotic to quantify surviving *E. coli* cells by counting cfu. An *in planta* bacterial competition assay was performed as described previously (29). Briefly, *A. tumefaciens* strains were transformed with gentamycin, resistance conferred by the pRL662 plasmid, or spectinomycin, resistance conferred by the pTrc200 plasmid, for selecting surviving cells. The attacker (*A*_600_ of 5) and target (*A*_600_ of 0.5) strains were mixed in ½ Murashige and Skoog medium (pH 5.7) at a 10:1 ratio and infiltrated into leaves of 6–7-week-old *Nicotiana benthamiana* plants by use of a needleless syringe. After a 24-h incubation at room temperature, the infiltrated spot was punched out, ground in 0.9% NaCl, serially diluted, and plated in triplicates on LB agar containing appropriate antibiotic to select for the target cells. All assays were performed with at least two independent experiments, each with two biological replicates, or three independent experiments, each with one or two biological replicates.

### P. aeruginosa type VI secretion and antibacterial competition assays

A *P. aeruginosa* type VI secretion assay was performed as described previously (41). In brief, *P. aeruginosa* strains harboring appropriate plasmids were grown in tryptone soy broth (TSB) overnight at 37 °C under agitation. Cells were harvested and subcultured to an *A*_600_ of 0.1, and then growth was continued in TSB to early stationary phase at 37 °C for 4–5 h to *A*_600_ of 5. Cells were separated from culture supernatants by centrifugation at 4000 × *g* at 4 °C. Cells were directly resuspended in 1 × SDS sample buffer. 10-Fold concentrated *P. aeruginosa* culture supernatant was prepared as follows. Proteins of the culture supernatant were precipitated using 6 m TCA (Sigma) at a final TCA concentration of 10%. Protein pellets were washed in 90% acetone, dried, and suspended in 1× SDS sample buffer; incubated at 96 °C for 20 min; and analyzed by SDS-PAGE. A *P. aeruginosa* antibacterial activity assay using *E. coli* as target cells was performed as described previously (42). In brief, overnight cultures of the indicated *P. aeruginosa* strains were incubated with overnight cultures of equivalent bacterial numbers of *E. coli* containing the plasmid pCR2.1 (carrying the *lacZ* gene) in a 1:1 ratio on LB agar for 5 h at 37 °C. In addition, *P. aeruginosa* and *E. coli* strains grown alone on LB agar for 5 h at 37 °C served as negative growth controls. Subsequently, patches of bacteria were collected and resuspended in LB broth, and dilution series ranging from 10^0^ to 10^−7^ were plated in triplicate on LB supplemented with 100 mg/ml 5-bromo-4-chloro-indolyl-β-d-galactopyranoside (X-gal; Invitrogen), allowing for colorimetric detection of *lacZ*-positive *E. coli* survivors. For quantitative analysis of the amount of *E. coli* survivors, the spots were harvested, serially diluted, and plated on LB agar plate containing X-gal and appropriate antibiotic to quantify surviving *E. coli* cells by counting. Data represent mean ± S.D. of all biological replicates.

### Yeast two-hybrid assay

The Matchmaker yeast two-hybrid system was used as instructed (Clontech, Mountain View, CA) and as described previously (30). Each of the plasmid pairs was co-transformed into *Saccharomyces cerevisiae* strain AH109. The transformants were selected by their growth on synthetic dextrose (SD) minimal medium lacking tryptophan (Trp) and leucine (Leu) (SD−WL medium). The positive interaction of expressed fusion proteins was then determined by their growth on SD lacking Trp, Leu, adenine (Ade), and histidine (His) (SD−WLHA medium) at 30 °C for at least 2 days.

### Total protein extraction from yeast

In brief, to prepare the total protein from yeast (43), overnight grown yeast strains harboring appropriate plasmids were subcultured at 28 °C in the same medium for further growth to *A*_600_ of 0.4–0.6. Yeast cells were harvested, 100 μl of protein extraction buffer containing 0.1% Nonidet P-40, 250 mm NaCl, 50 mm Tris-HCl (pH 7.5), 5 mm EDTA (pH 8.0), 1 mm DTT, 2× protease inhibitor mixtures (Roche Applied Science), 4 mm phenylmethylsulfonyl fluoride, and 50 μl of acid-washed glass beads (Sigma-Aldrich) were added. The cells were broken by vortex at the highest speed for 30 s, and then tubes were placed on ice for 30 s. The same procedure was repeated six times to make sure cells had been completely broken. The supernatant above the glass beads was collected (the first cell extract). Then 50 μl of protein extraction buffer was added to wash the tube containing broken cells and glass beads by vortexing at the highest speed for 30 s. The supernatant above the glass beads was collected again (the second cell extract). The two extracts were mixed together, and the final protein extract was centrifuged at 13,000 rpm for 5 min at 4 °C; the resulting supernatant was collected, and protein concentration was measured. An equal volume of 2× SDS loading buffer was added to the final protein sample, incubated at 96 °C for 20 min, and analyzed by SDS-PAGE.

### Bacterial two-hybrid assay

The BTH assay was performed as described previously (44). In brief, DNA fragments encoding the protein of interest were amplified by PCR, adding appropriate restriction sites into the primers, using *P. aeruginosa* PAK genomic DNA. DNA fragments encoding the proteins or protein domains of interest were cloned into plasmids pKT25 and pUT18C, which each encode for complementary fragments of the adenylate cyclase enzyme, as described previously (44), resulting in constructs expressing an N-terminal fusion of the protein of interest with the T25 or T18 subunit of adenylate cyclase. Recombinant pKT25 and pUT18C plasmids were co-transformed into the *E. coli* DHM1 strain, which is devoid of adenylate cyclase, and transformants were spotted onto LB agar plates (Difco) supplemented with 40 mg/ml X-gal in the presence of 100 mg/ml ampicillin, 50 mg/ml kanamycin, and 1 mm IPTG. Positive interactions were identified as blue colonies on LB agar plates containing X-gal after a 48-h incubation at 30 °C. The experiments were performed at least in duplicate, and a representative result is shown.

### β-gal assay

For quantitative analysis of BTH interactions, β-gal activity of co-transformants scraped from LB agar plates containing X-gal was measured as described previously, and activity was calculated in Miller units (45, 46).

### Statistical analysis

Data represent mean ± S.E. of all biological replicates. Statistics were calculated by one-way analysis of variance and Tukey's honestly significance difference test (http://astatsa.com/OneWay_Anova_with_TukeyHSD/),^5^ and significant differences are indicated (*p* < 0.01 or *p* < 0.05).

### Dephosphorylation and Phos-tag SDS-PAGE analyses

Dephosphorylation analysis by calf intestinal alkaline phosphatase (CIAP) was performed according to the user manual (New England Biolabs, Beverly, MA) with minor modifications as described previously (22). Equal amounts of Ni-NTA resins with purified TssL-His isolated from various *A. tumefaciens* strains were resuspended in 1× CIAP buffer containing 100 mm NaCl, 50 mm Tris-HCl (pH 7.9), 10 mm MgCl_2_, 1 mm DTT, and 1× protease inhibitor mixture (EDTA-free) with CIAP at 1 unit/μg of protein. The protein samples treated with or without CIAP were incubated at 37 °C for 90 min. An equal volume of 2× SDS loading buffer was added and incubated at 96 °C for 20 min and analyzed by Phos-tag SDS-PAGE. The Phos-tag SDS-PAGE analysis was performed according to the user manual for Phos-tag acrylamide AAL-107 (Wako Pure Chemical Industries, Osaka, Japan) with minor modifications as described previously (22). Protein samples were separated on 7% polyacrylamide gels containing 0.35 m BisTris-HCl (pH 6.8), 35 μm Phos-tag acrylamide AAL-107, and 100 μm ZnCl_2_, with electrophoresis conducted at 40 mA/gel under a maximum voltage of 90 V in a cold room. After electrophoresis, Phos-tag gels were washed with transfer buffer (25 mm Tris, 192 mm glycine, 20% methanol) containing 1 mm EDTA for 15 min with gentle shaking followed by a second wash in transfer buffer without EDTA for 15 min. The gels were washed with transfer buffer containing 1% SDS for 15 min before transfer to polyvinylidene difluoride membranes with a submarine blotting apparatus.

### Protein purification and gel filtration analysis

N-terminal His-tagged TagF aa 1–214 proteins were expressed in *E. coli* BL21 (DE3) cells with the plasmid pET28a(+)-TagF 1–214. *E. coli* cells were grown in LB medium in the presence of kanamycin (20 μg/ml) at 37 °C until the cell density reached an *A*_600_ of 0.6–0.8. The cultures were induced with 1 mm IPTG for 16–20 h at 16 °C to induce production of His-tagged TagF aa 1–214 proteins. The cells were harvested by centrifugation followed by snap-freezing by liquid nitrogen and stored at −80 °C. Frozen bacterial pellets were resuspended with the lysis buffer (150 mm sodium chloride, 10 mm imidazole, and 50 mm Tris-HCl, pH 7.5), and then the cells were lysed by a microfluidizer. The cell lysate was clarified by centrifugation at 20,000 rpm for 40 min at 4 °C, and the supernatant was loaded onto an Ni-NTA column (GE Healthcare) pre-equilibrated with the lysis buffer. The column was washed with the washing buffer (150 mm sodium chloride, 80 mm imidazole, and 50 mm Tris-HCl, pH 7.5), and the bound protein was eluted by the elution buffer (150 mm sodium chloride, 300 mm imidazole, and 50 mm Tris-HCl, pH 7.5). The TagF aa 1–214 proteins were further analyzed by size-exclusion chromatography using a Superdex 75 16 × 60 column through a fast protein LC (FPLC) system (GE Healthcare). The column was equilibrated with 2 column volumes of buffer containing 50 mm Tris-HCl (pH 7.5) and 150 mm NaCl. To determine molecular weight, a parallel column was run with protein standards. The elution profiles were recorded as absorbance at 280 nm.

### Western blot analysis

Western blot analysis was performed as described previously (40) with the primary polyclonal pTssL antibody produced in this study and those against proteins PpkA, TagF-PppA, TssK, Fha, TssC_41_, TssB, TssA, ClpV, Tae, VgrGs, and RpoA (30), TssL (4), TssM (4), Hcp (27), ActC (47), Tde1 (29), *P. aeruginosa* Hcp1, TssB1, TagJ1, and Tse3 (44), polyclonal antibodies against His (Sigma-Aldrich), monoclonal antibodies against HA (Sigma-Aldrich), the β subunit of RNA polymerase (Neoclone), c-Myc (Sigma-Aldrich), or Strep (IBA-Life Sciences, Goettingen, Germany), followed by incubation with a secondary antibody, horseradish peroxidase–conjugated goat anti-rabbit IgG (Chemichem) and detection by use of the Western Lightning System (PerkinElmer Life Sciences). Chemiluminescent bands were visualized on X-ray film (GE Healthcare).

## Author contributions

J.-S. L. and E.-M. L. conceptualization; J.-S. L., P. P., and H.-H. W. data curation; J.-S. L., P. P., H.-H. W., M.-D. T., A. F., and E.-M. L. investigation; J.-S. L. and E.-M. L. writing-original draft; J.-S. L., P. P., H.-H. W., M.-D. T., A. F., and E.-M. L. writing-review and editing; M.-D. T., A. F., and E.-M. L. supervision; M.-D. T., A. F., and E.-M. L. funding acquisition.

## Supplementary Material

Supporting Information

## Footnotes

5 Please note that the JBC is not responsible for the long-term archiving and maintenance of this site or any other third party hosted site.

## Footnotes

4 The abbreviations used are:

T6SS: type VI secretion system
FHA: forkhead-associated
TPP: threonine phosphorylation pathway
aa: amino acids
Ni-NTA: nickel-nitrilotriacetic acid
pTssL: phosphorylated TssL
YTH: yeast two-hybrid
BTH: bacterial two-hybrid
LB: lysogeny broth
IPTG: isopropyl β-d-thiogalactopyranoside
cfu: colony-forming units
TSB: tryptone soy broth
X-gal: 5-bromo-4-chloro-3-indolyl β-d-galactoside
CIAP: calf intestinal alkaline phosphatase
BisTris: 2-[bis(2-hydroxyethyl)amino]-2-(hydroxymethyl)propane-1,3-diol
TagF: type VI secretion–associated gene F.
