# Supplementary material for: TagF-mediated repression of bacterial type VI secretion systems involves a direct interaction with the cytoplasmic protein Fha
Source: J Biol Chem. 2018 Mar 29;293(23):8829–42. doi: 10.1074/jbc.RA117.001618 (PMC5995506; doi:10.1074/jbc.RA117.001618)
Supplement: Supporting Information [file supp_293_23_8829__index.html]

TagF-mediated repression of bacterial type VI secretion systems involves a direct interaction with the cytoplasmic protein Fha — TagF-mediated T6SS repression — TagF-mediated repression of bacterial type VI secretion systems involves a direct interaction with the cytoplasmic protein Fha — TagF-mediated T6SS repression — Supporting Information 

# TagF-mediated repression of bacterial type VI secretion systems involves a direct interaction with the cytoplasmic protein Fha

## Supporting Information

- Table S1 - Bacterial strains and Plasmids
- Table S2 - Primers
- Supporting information S1 - Supporting materials and methods
- Supporting figures - Supporting figures S1-S4
