# Supplementary material for: TagF-mediated repression of bacterial type VI secretion systems involves a direct interaction with the cytoplasmic protein Fha
Source: J Biol Chem. 2018 Mar 29;293(23):8829–42. doi: 10.1074/jbc.RA117.001618 (PMC5995506; doi:10.1074/jbc.RA117.001618)
Supplement: Supporting Information [file supp_RA117.001618_134892_1_supp_88861_p559vk.doc]

**Table S1. Bacterial strains and plasmids**

| Strain /plasmid | Relevant characteristics | Source/ reference |
| --- | --- | --- |
| *A. tumefaciens* |  |  |
| C58 | Wild type virulent strain containing nopaline-type Ti plasmid pTiC58 | Eugene Nester |
| EML1060 | *ppkA*(*atu4330*) in frame deletion mutant, C58Δ*ppkA* |  |
| EML1063 | *tagF-pppA* (*atu4331*) in frame deletion mutant, C58Δ*tagF*-*pppA* |  |
| EML4307 | *ppkA* and *tagF-pppA* in-frame deletion mutant, C58Δ*ppkA*Δ*tagF-pppA* | This study |
| EML1073 | *tssL* (*atu4333*) in-frame deletion mutant, C58Δ*tssL* |  |
| EML3561 | Deletion from *atu3639* to *atu3640*, *atu4350* to *atu4351*, and *atu4346* to *atu4347*, C58Δ*3TIs* |  |
| EML4515 | *tagF-pppA* with G22A and K23A substitutions, C58*tagF^GK^-pppA* | This study |
| EML4517 | *tagF-pppA* with D44A and W46A substitutions, C58*tagF^DW^-pppA* | This study |
| EML4519 | *tagF-pppA* with S93A, D95A and R99A substitutions, C58*tagF^SDR^-pppA* | This study |
| *P. aeruginosa* |  |  |
| PAKΔ*retS* | In-frame deletion of *retS* (PA4856) in PAK |  |
| PAKΔ*retS*Δ*H1* | Deletion of *retS* and H1-T6SS cluster in PAK |  |
| PAKΔ*retS*Δ*tssB1* | In-frame deletion of *retS* and *tssB1* (PA0083) in PAK |  |
| *E. coli* |  |  |
| DH10B | Host for DNA cloning | Invitrogen |
| DH5α | Host for DNA cloning | Laboratory collection |
| BL21 (DE3) | Host for overexpressing genes driven by the T7 promoter |  |
| *S. cerevisiae* |  |  |
| AH109 | Host for yeast two-hybrid analysis | Clontech |
| Plasmids |  |  |
| pRL662 | Gm^R^, broad-host range vector derived from pBBR1MCS-2 |  |
| pJQ200KS | Gm^R^, suicide plasmid containing Gm^r^ and *sacB* gene for selection of double crossover |  |
| pET28a(+) | Km^R^, *E. coli* overexpression vector to produce N or C-terminal His-tagged protein | Novagen |
| pTrc200 | Sp^R^ , pVS1 origin *lacI*^q^, *trc* promoter expression vector |  |
| pCR2.1 | Ap^R^, Km^R^, TA cloning vector | Invitrogen |
| pGADT7 | Ap^R^, AD vector used in yeast two-hybrid assay | Clontech |
| pGBKT7 | Km^R^, DNA-BD vector used in yeast-two hybrid assay | Clontech |
| pKT25 | Km^R^, BTH vector for fusion of target proteins to *B. pertussis cya* gene T25 fragment; P_lac_::*cya*^1–675^ p15ori |  |
| pUT18C | Ap^R^, BTH vector for fusion of target proteins to *B. pertussis cya* gene T18 fragment; P_lac_::*cya*^675–1197^ pUCori |  |
| pTagF^Pa^ | Gm^R^, pRL662 expressing *P. aeruginosa* TagF driven by *lacZp* | This study |
| pTagF^Pa^-Strep | Gm^R^, pRL662 expressing *P. aeruginosa* TagF-Strep fusion protein driven by *lacZp* | This study |
| pTagF^Pa^-^GK^-Strep | Gm^R^, pRL662 expressing *P. aeruginosa* TagF-Strep fusion protein with G8A and K9A substitutions driven by *lacZp* | This study |
| pTagF^Pa^-^SDR^-Strep | Gm^R^, pRL662 expressing *P. aeruginosa* TagF-Strep fusion protein with S79A, D81A, and R85A substitutions driven by *lacZp* | This study |
| pTssL-His | Gm^R^, pRL662 expressing TssL-His fusion protein driven by *lacZp* |  |
| pTrc-TagF-PppA | Sp^R^, pTrc200 expressing TagF-PppA full-length protein | This study |
| pTrc-TagF | Sp^R^, pTrc200 expressing TagF domain (1-230 a.a.) | This study |
| pTrc-TagF-Strep | Sp^R^, pTrc200 expressing TagF-Strep fusion protein | This study |
| pTrc-TagF^GK^-Strep | Sp^R^, pTrc200 expressing TagF-Strep fusion protein with G22A and K23A substitutions | This study |
| pTrc-TagF^DF^-Strep | Sp^R^, pTrc200 expressing TagF-Strep fusion protein with D29A and F30A substitutions | This study |
| pTrc-TagF^DW^-Strep | Sp^R^, pTrc200 expressing TagF-Strep fusion protein with D44A and W46A substitutions | This study |
| pTrc-TagF^SDR^-Strep | Sp^R^, pTrc200 expressing TagF-Strep fusion protein with S93A, D95A and R99A substitutions | This study |
| pTrc-TagF^FD^-Strep | Sp^R^, pTrc200 expressing TagF-Strep fusion protein with F141A and D142A substitutions | This study |
| pTrc-PppA | Sp^R^, pTrc200 expressing PppA domain (231-471 a.a.) | This study |
| pTrc-Fha1^Pa^-HA | Sp^R^, pTrc200 expressing *P. aeruginosa* Fha1-HA fusion protein | This study |
| pGBKT7-TagF-PppA | Km^R^, DNA-BD vector expressing TagF-PppA | This study |
| pGBKT7-TagF | Km^R^, DNA-BD vector expressing TagF domain (1-230 a.a.) | This study |
| pGBKT7-TagF^GK^ | Km^R^, DNA-BD vector expressing TagF domain (1-230 a.a.) with G22A and K23A substitutions | This study |
| pGBKT7-TagF^DF^ | Km^R^, DNA-BD vector expressing TagF domain (1-230 a.a.) with D29A and F30A substitutions | This study |
| pGBKT7-TagF^DW^ | Km^R^, DNA-BD vector expressing TagF domain (1-230 a.a.) with D44A and W46A substitutions | This study |
| pGBKT7-TagF^SDR^ | Km^R^, DNA-BD vector expressing TagF domain (1-230 a.a.) with S93A, D95A and R99A substitutions | This study |
| pGBKT7-TagF^FD^ | Km^R^, DNA-BD vector expressing TagF domain (1-230 a.a.) with F141A and D142A substitutions | This study |
| pGBKT7-TagF^Pa^ | Km^R^, DNA-BD vector expressing *P. aeruginosa* TagF | This study |
| pGBKT7-TagF^Pa-GK^ | Km^R^, DNA-BD vector expressing *P. aeruginosa* TagF with G8A and K9A substitutions | This study |
| pGBKT7-TagF^Pa-SDR^ | Km^R^, DNA-BD vector expressing *P. aeruginosa* TagF with S79A, D81A, and R85A substitutions | This study |
| pGBKT7-Fha1^Pa^ | Km^R^, DNA-BD vector expressing *P. aeruginosa* Fha1 | This study |
| pGBKT7-53 | Km^R^, DNA-BD vector expressing murine p53 | Clontech |
| pGADT7-TagF | AP^R^, AD vector expressing TagF domain (1-230 a.a.) | This study |
| pGADT7-Fha | AP^R^, AD vector expressing Fha | This study |
| pGADT7-TagF^Pa^ | AP^R^, AD vector expressing *P. aeruginosa* TagF | This study |
| pGADT7-Fha1^Pa^ | AP^R^, AD vector expressing *P. aeruginosa* Fha1 | This study |
| pGADT7-T | Ap^R^, AD vector expressing SV40 large T-antigen | Clontech |
| pKT25-TagF^Pa^ | Km^R^, fusion of *tagF^Pa^* to *cya* gene T18 fragment in pKT25 | This study |
| pKT25-Fha1^Pa^ | Km^R^, fusion of *fha1^Pa^* to *cya* gene T18 fragment in pKT25 | This study |
| pUT18C-TagF^Pa^ | Ap^R^, fusion of *tagF^Pa^* to *cya* gene T25 fragment in pUT18C | This study |
| pUT18C-Fha1^Pa^ | Ap^R^, fusion of *fha1^Pa^* to *cya* gene T25 fragment in pUT18C | This study |
| pJQ200KS-Δ*ppkA*Δ*tagF-pppA* | Gm^R^, used in generating *ppkA* and *tagF-pppA* double deletion mutant of *A. tumefaciens* C58 | This study |
| pJQ200KS- *tagF^GK^-pppA* | Gm^R^, used in generating *tagF-pppA* with G22A and K23A substitutions of *A. tumefaciens* C58 | This study |
| pJQ200KS- *tagF^DW^-pppA* | Gm^R^, used in generating *tagF-pppA* with D44A and W46A substitutions of *A. tumefaciens* C58 | This study |
| pJQ200KS- *tagF^SDR^-pppA* | Gm^R^, used in generating *tagF-pppA* with S93A, D95A, and R99A substitutions of *A. tumefaciens* C58 | This study |
| pET28a(+)-*tagF* 1-214 | Km^R^, pET28a(+) expressing N-terminal His-tagged TagF 1-214 aa protein | This study |

1. Lin, J. S., Ma, L. S., and Lai, E. M. (2013) Systematic Dissection of the Agrobacterium Type VI Secretion System Reveals Machinery and Secreted Components for Subcomplex Formation. *PLoS One* **8**, e67647

2. Ma, L. S., Lin, J. S., and Lai, E. M. (2009) An IcmF family protein, ImpLM, is an integral inner membrane protein interacting with ImpKL, and its walker a motif is required for type VI secretion system-mediated Hcp secretion in Agrobacterium tumefaciens. *J Bacteriol* **191**, 4316-4329

3. Ma, L. S., Hachani, A., Lin, J. S., Filloux, A., and Lai, E. M. (2014) Agrobacterium tumefaciens deploys a superfamily of type VI secretion DNase effectors as weapons for interbacterial competition in planta. *Cell Host Microbe* **16**, 94-104

4. Goodman, A. L., Kulasekara, B., Rietsch, A., Boyd, D., Smith, R. S., and Lory, S. (2004) A signaling network reciprocally regulates genes associated with acute infection and chronic persistence in Pseudomonas aeruginosa. *Dev Cell* **7**, 745-754

5. Hachani, A., Lossi, N. S., and Filloux, A. (2013) A visual assay to monitor T6SS-mediated bacterial competition. *Journal of visualized experiments : JoVE*, e50103

6. Planamente, S., Salih, O., Manoli, E., Albesa-Jove, D., Freemont, P. S., and Filloux, A. (2016) TssA forms a gp6-like ring attached to the type VI secretion sheath. *EMBO J* **35**, 1613-1627

7. Studier, F. W., Rosenberg, A. H., Dunn, J. J., and Dubendorff, J. W. (1990) Use of T7 RNA polymerase to direct expression of cloned genes. *Methods Enzymol* **185**, 60-89

8. Vergunst, A. C., Schrammeijer, B., den Dulk-Ras, A., de Vlaam, C. M., Regensburg-Tuink, T. J., and Hooykaas, P. J. (2000) VirB/D4-dependent protein translocation from Agrobacterium into plant cells. *Science* **290**, 979-982

9. Quandt, J., and Hynes, M. F. (1993) Versatile suicide vectors which allow direct selection for gene replacement in gram-negative bacteria. *Gene* **127**, 15-21

10. Schmidt-Eisenlohr, H., Domke, N., and Baron, C. (1999) TraC of IncN plasmid pKM101 associates with membranes and extracellular high-molecular-weight structures in Escherichia coli. *J Bacteriol* **181**, 5563-5571

11. Karimova, G., Pidoux, J., Ullmann, A., and Ladant, D. (1998) A bacterial two-hybrid system based on a reconstituted signal transduction pathway. *Proc Natl Acad Sci U S A* **95**, 5752-5756

12. Lin, J. S., Wu, H. H., Hsu, P. H., Ma, L. S., Pang, Y. Y., Tsai, M. D., and Lai, E. M. (2014) Fha interaction with phosphothreonine of TssL activates type VI secretion in Agrobacterium tumefaciens. *PLoS Pathog* **10**, e1003991
