# Supplementary material for: TagF-mediated repression of bacterial type VI secretion systems involves a direct interaction with the cytoplasmic protein Fha
Source: J Biol Chem. 2018 Mar 29;293(23):8829–42. doi: 10.1074/jbc.RA117.001618 (PMC5995506; doi:10.1074/jbc.RA117.001618)
Supplement: Supporting Information [file supp_RA117.001618_134892_1_supp_88862_p559vk.doc]

Table S2. Primer information

| Primer | Plasmids | Sequence (5 '-3')^a^ | Source / reference |
| --- | --- | --- | --- |
| TagF-PppA F-BamHI | pTrc-TagF | 5'-CGGGATCCTTATGATGAAGGCAAGCACG-3' |  |
| TagF N1-230 R-XbaI |  | 5'-GCTCTAGACTATGTCTTCTCCTGCACCGTTGC-3' | This study |
| TagF-PppA F-BamHI | pTrc-TagF-Strep | 5'-CGGGATCCTTATGATGAAGGCAAGCACG-3' |  |
| TagF N1-230-Strep R-XbaI |  | 5'-GCTCTAGACTACTTTTCGAACTGCGGGTGGCTCCATGTCTTCTCCTGCACCGTTGC-3' | This study |
| TagF-PppA F-BamHI | pTrc-TagF^GK^-Strep | 5'-CGGGATCCTTATGATGAAGGCAAGCACG-3' |  |
| TagF N1-230-Strep R-XbaI |  | 5'-GCTCTAGACTACTTTTCGAACTGCGGGTGGCTCCATGTCTTCTCCTGCACCGTTGC-3' | This study |
| TagF GK-1 |  | 5'-CCATGGCTGGGGACC**GC**G**G**CGAAGAAACCGATGC-3' | This study |
| TagF GK-2 |  | 5'-GCATCGGTTTCTTCG**C**C**GC**GGTCCCCAGCCATGG-3' | This study |
| TagF-PppA F-BamHI | pTrc-TagF^DF^-Strep | 5'-CGGGATCCTTATGATGAAGGCAAGCACG-3' |  |
| TagF N1-230-Strep R-XbaI |  | 5'-GCTCTAGACTACTTTTCGAACTGCGGGTGGCTCCATGTCTTCTCCTGCACCGTTGC-3' | This study |
| TagF DF-1 |  | 5'-GAGCCCATCGGAAATG**GC**A**G**CGCCATGGCTGGGGAC-3' | This study |
| TagF DF-2 |  | 5'-GTCCCCAGCCATGGCG**C**T**GC**CATTTCCGATGGGCTC-3' | This study |
| TagF-PppA F-BamHI | pTrc-TagF^DW^-Strep | 5'-CGGGATCCTTATGATGAAGGCAAGCACG-3' |  |
| TagF N1-230-Strep R-XbaI |  | 5'-GCTCTAGACTACTTTTCGAACTGCGGGTGGCTCCATGTCTTCTCCTGCACCGTTGC-3' | This study |
| TagF DW-1 |  | 5'-CATGCCGGATC**GC**ATCGCGTCG**G**CGAAGGTAGCGATCA-3' | This study |
| TagF DW-2 |  | 5'-TGATCGCTACCTTCG**C**CGAC**GC**GATGCGATCCGGCATG-3' | This study |
| TagF-PppA F-BamHI | pTrc-TagF^SDR^-Strep | 5'-CGGGATCCTTATGATGAAGGCAAGCACG-3' |  |
| TagF N1-230-Strep R-XbaI |  | 5'-GCTCTAGACTACTTTTCGAACTGCGGGTGGCTCCATGTCTTCTCCTGCACCGTTGC-3' | This study |
| TagF SDR-1 |  | 5'-GGATATTTA**GC**GCCCACCCG**GG**CGGCG**GC**CGGCACCAGCACG-3' | This study |
| TagF SDR-2 |  | 5'-CGTGCTGGTGCCG**GC**CGCCG**CC**CGGGTGGGC**GC**TAAATATCC-3' | This study |
| TagF-PppA F-BamHI | pTrc-TagF^FD^-Strep | 5'-CGGGATCCTTATGATGAAGGCAAGCACG-3' |  |
| TagF N1-230-Strep R-XbaI |  | 5'-GCTCTAGACTACTTTTCGAACTGCGGGTGGCTCCATGTCTTCTCCTGCACCGTTGC-3' | This study |
| TagF FD-1 |  | 5'-CATTAAACCGGGACACG**G**CG**GC**ATCGCCGGTCATCGAT-3' | This study |
| TagF FD-2 |  | 5'-ATCGATGACCGGCGAT**GC**CG**C**CGTGTCCCGGTTTAATG-3' | This study |
| TagF^Pa^ F-XhoI | pTagF^Pa^ | 5'-CCGCTCGAGCCAGCATCGCCTGCGAGCTGC-3' | This study |
| TagF^Pa^ R-XbaI |  | 5'-GCTCTAGACGACCTGTAGTAGCTGACTGAG-3' | This study |
| TagF^Pa^ F-XhoI | pTagF^Pa^-Strep | 5'-CCGCTCGAGCCAGCATCGCCTGCGAGCTGC-3' | This study |
| TagF^Pa^ Strep R-XbaI |  | 5'-GCTCTAGACTACTTTTCGAACTGCGGGTGGCTCCAACCGGGTATGCCGGGAAAGAGC-3' | This study |
| TagF^Pa^ F-XhoI | pTagF^Pa-GK^-Strep | 5'-CCGCTCGAGCCAGCATCGCCTGCGAGCTGC-3' | This study |
| TagF^Pa^ Strep R-XbaI |  | 5'-GCTCTAGACTACTTTTCGAACTGCGGGTGGCTCCAACCGGGTATGCCGGGAAAGAGC-3' | This study |
| TagF^Pa^ GK-1 |  | 5'-CCGCGGCCGGCCAGC**GC**G**G**CGTAGAAACCGACGC-3' | This study |
| TagF^Pa^ GK-2 |  | 5'-GCGTCGGTTTCTACG**C**C**GC**GCTGGCCGGCCGCGG-3' | This study |
| TagF^Pa^ F-XhoI | pTagF^Pa-SDR^-Strep | 5'-CCGCTCGAGCCAGCATCGCCTGCGAGCTGC-3' | This study |
| TagF^Pa^ Strep R-XbaI |  | 5'-GCTCTAGACTACTTTTCGAACTGCGGGTGGCTCCAACCGGGTATGCCGGGAAAGAGC-3' | This study |
| TagF^Pa^ SDR-1 |  | 5'-GGGAAATAG**GC**ACCGACCCGG**G**CGATG**GC**CGGCATCACCACT-3' | This study |
| TagF^Pa^ SDR-2 |  | 5'-AGTGGTGATGCCG**GC**CATCG**C**CCGGGTCGGT**GC**CTATTTCCC-3' | This study |
| PppA C231-471-BamHI | pTrc-PppA | 5'-CGGGATCCATGAAACAGCAAATACCGCCCGTG-3' | This study |
| TagF-PppA R-XbaI |  | 5'-GCTCTAGAGGATAGGCAGGCTCATCAAG-3' |  |
| TagF-PppA F-BamHI | pTrc-TagF-PppA | 5'-CGGGATCCTTATGATGAAGGCAAGCACG-3' |  |
| TagF-PppA R-XbaI |  | 5'-GCTCTAGAGGATAGGCAGGCTCATCAAG-3' |  |
| Fha1^Pa^ F-NcoI | pTrc-Fha1^Pa^-HA | 5'-CATGCCATGGCCTGGATGCGAACCGAAATCC-3' | This study |
| Fha1^Pa^ HA R-XbaI |  | 5'-GCTCTAGATCAAGCGTAATCTGGAACATCGTATGGGTAGGAACGCCGTAGTCGAGCGCTG-3' | This study |
| TagF-PppA F | pGBKT7-TagF-PppA | 5'-TGGCCGATCAGGCATCAAGG-3' |  |
| TagF-PppA R-BamHI |  | 5'-CGGGATCCGCCTTGCTCACGCCGGTTTC-3' | This study |
| TagF-PppA F | 1. pGBKT7-TagF 2. pGBKT7-TagF^GK^ (pTrc-TagF^GK^-Strep as template) 3. pGBKT7-TagF^DF^   (pTrc-TagF^DF^-Strep as template)   1. pGBKT7-TagF^DW^   (pTrc-TagF^DW^-Strep as template)   1. pGBKT7-TagF^SDR^   (pTrc-TagF^SDR^-Strep as template)   1. pGBKT7-TagF^FD^   (pTrc-TagF^FD^-Strep as template)   1. pGADT7-TagF | 5'-TGGCCGATCAGGCATCAAGG-3' |  |
| TagF R-BamHI |  | 5'-CGGGATCCTGTCTTCTCCTGCACCGTTGC-3' | This study |
| Fha F-NdeI | pGADT7-Fha | 5'-GAACATATGAAGCTTGCACTCAAGAACAC-3' |  |
| Fha R-BamHI |  | 5'-CGGGATCCTGTCTCATCGTGGTTGTTTACC-3' | This study |
| TagF^Pa^ F | 1. pGBKT7-TagF^Pa^ 2. pGBKT7-TagF^Pa-GK^ (pTrc-TagF^Pa-GK^-Strep as template) 3. pGBKT7-TagF^Pa-SDR^ (pTrc-TagF^Pa-SDR^-Strep as template) 4. pGADT7-TagF^Pa^ | 5'-TGTTGAACAGCGTCGGTTTCTACG-3' | This study |
| TagF^Pa^ R-BamHI |  | 5'-CGGGATCCACCGGGTATGCCGGGAAAGAGC-3' | This study |
| Fha1^Pa^ F | 1. pGBKT7-Fha1^Pa^ 2. pGADT7-Fha1^Pa^ | 5'-TGCCGCTGCGATTGACCATCAC-3' | This study |
| Fha1^Pa^ R-BamHI |  | 5'-CGGGATCCGGAACGCCGTAGTCGAGCGCTG-3' | This study |
| TagF^Pa^ F-XbaI | 1. pKT25-TagF^Pa^ 2. pUT18C-TagF^Pa^ | 5'-GCGCGTCTAGAGTTGAACAGCGTCGGTTTCTACG-3' | This study |
| TagF^Pa^ R-EcoRI |  | 5'-GCGCGGAATTCTTAACCGGGTATGCCGGGA-3' | This study |
| Fha1^Pa^ F-XbaI | 1. pKT25-Fha1^Pa^ 2. pUT18C-Fha1^Pa^ | 5'-GTTAGTCTAGAGATGCCGCTGCGATTGACCAT-3' | This study |
| Fha1^Pa^ R-BamHI |  | 5'-AATACGGATCCTCAGGAACGCCGTAGTCGAG-3' | This study |
| PpkA 2F-BamHI | pJQ200KS-Δ*ppkA*Δ*tagF-pppA* | 5'-CGGGATCCCTGTAGCGCCGGCGTCAGTTG-3' |  |
| PpkA 2R-XmaI |  | 5'-TCCCCCCGGGCCCGTCAGGAGCGTGTACTTG-3' |  |
| TagF-PppA 1F-XbaI |  | 5'-GCTCTAGAGCCCAGTTCGAAAATGCCGAC-3' |  |
| TagF-PppA 1R-BamHI |  | 5'-CGGGATCCATCGGCCATCAGTTGCGATTG-3' |  |
| TagF-PppA 1F-XbaI | pJQ200KS-*tagF^GK^-pppA* | 5'-GCTCTAGAGCCCAGTTCGAAAATGCCGAC-3' |  |
| TagF-PppA 2R-XmaI |  | 5'-TCCCCCCGGGCGAAGGATCGAGATCACCTGC-3' |  |
| TagF GK-1 |  | 5'-CCATGGCTGGGGACC**GC**G**G**CGAAGAAACCGATGC-3' | This study |
| TagF GK-2 |  | 5'-GCATCGGTTTCTTCG**C**C**GC**GGTCCCCAGCCATGG-3' | This study |
| TagF-PppA 1R |  | 5'-GATCGATGTGCCACCAGAGG-3' | This study |
| TagF-PppA 1F-XbaI | pJQ200KS-*tagF^DW^-pppA* | 5'-GCTCTAGAGCCCAGTTCGAAAATGCCGAC-3' |  |
| TagF-PppA 2R-XmaI |  | 5'-TCCCCCCGGGCGAAGGATCGAGATCACCTGC-3' |  |
| TagF DW-1 |  | 5'-GAGCCCATCGGAAATG**GC**A**G**CGCCATGGCTGGGGAC-3' | This study |
| TagF DW-2 |  | 5'-GTCCCCAGCCATGGCG**C**T**GC**CATTTCCGATGGGCTC-3' | This study |
| TagF-PppA 1R |  | 5'-GATCGATGTGCCACCAGAGG-3' | This study |
| TagF-PppA 1F-XbaI | pJQ200KS-*tagF^SDR^-pppA* | 5'-GCTCTAGAGCCCAGTTCGAAAATGCCGAC-3' |  |
| TagF-PppA 2R-XmaI |  | 5'-TCCCCCCGGGCGAAGGATCGAGATCACCTGC-3' |  |
| TagF SDR-1 |  | 5'-GGATATTTA**GC**GCCCACCCG**GG**CGGCG**GC**CGGCACCAGCACG-3' | This study |
| TagF SDR-2 |  | 5'-CGTGCTGGTGCCG**GC**CGCCG**CC**CGGGTGGGC**GC**TAAATATCC-3' | This study |
| TagF-PppA 1R |  | 5'-GATCGATGTGCCACCAGAGG-3' | This study |
| TagF 1-214 F | pET28a(+)-*tagF* 1-214 | 5'-CAGGCAAAGGCGACGAGTAACAAAGCCCGAAAG-3' | This study |
| TagF 1-214 R |  | 5'-CTTTCGGGCTTTGTTACTCGTCGCCTTTGCCTG-3' | This study |

a: Restriction enzyme sites are underlined, and mutated sequences are indicated by bold type.

1. Lin, J. S., Wu, H. H., Hsu, P. H., Ma, L. S., Pang, Y. Y., Tsai, M. D., and Lai, E. M. (2014) Fha interaction with phosphothreonine of TssL activates type VI secretion in Agrobacterium tumefaciens. *PLoS Pathog* **10**, e1003991

2. Lin, J. S., Ma, L. S., and Lai, E. M. (2013) Systematic Dissection of the Agrobacterium Type VI Secretion System Reveals Machinery and Secreted Components for Subcomplex Formation. *PLoS One* **8**, e67647
