# Supplementary material for: TagF-mediated repression of bacterial type VI secretion systems involves a direct interaction with the cytoplasmic protein Fha
Source: J Biol Chem. 2018 Mar 29;293(23):8829–42. doi: 10.1074/jbc.RA117.001618 (PMC5995506; doi:10.1074/jbc.RA117.001618)
Supplement: Supporting Information [file supp_RA117.001618_134892_1_supp_91518_p5bxyg.pdf]

Supporting Information S1

The growth conditions. *A. tumefaciens* was grown at 25 °C in 523 (1), whereas LB (2) were routinely used for *E. coli* and *P. aeruginosa* strains at 37 °C unless indicated. The plasmids were maintained by the addition of 50 μg/mL gentamycin (Gm) and 200 μg/mL spectinomycin (Sp) for *A. tumefaciens* and 100 μg/mL ampicillin (Ap), 100 μg/mL spectinomycin (Sp), 20 μg/mL kanamycin (Km), and 50 μg/mL Gm for *E. coli*, and 50 μg/mL Gm, and 2000 μg/mL Sp for *P. aeruginosa.* Growth conditions are as previously described (3,4).

Plasmid construction and generation of in-frame deletion mutants. Plasmid pJQ200KS-Δ*ppkA*Δ*tagF-pppA* (Supplementary Table S1) was created by ligating the *Xba*I/*Bam*HI-digested PCR product 1 (~500 bp DNA fragment upstream of *tagF-pppA* gene) and the *BamH*I/*Xma*I-digested PCR product 2 (~500 bp DNA fragment downstream of *ppkA* gene) into *Xba*I/*Xma*I sites of pJQ200KS (5) and used to generate the *ppkA* and t*agF-pppA* genes deletion mutant (Supplementary Tables S1 and S2). The resulting strain was confirmed by PCR and designated as EML4307 (Δ*ppkA*Δ*tagF-pppA*).

To construct the plasmids for expressing proteins in *A. tumefaciens*, each DNA fragment containing the ribosomal-binding sequence (RBS) and ORF (with stop codon) of *tagF-pppA*, *tagF*, *tagF-Strep*, $tagF^{GK}$*-Strep*, $tagF^{DF}$*-Strep*, $tagF^{DW}$*-Strep*, $tagF^{SDR}$*-Strep*, $tagF^{FD}$*-Strep*, and *pppA* were PCR-amplified with primers described in Supplementary Table S2 and respectively cloned into pTrc200 (6) with appropriate enzyme sites to create the plasmids pTrc-TagF-PppA, pTrc-TagF, pTrc-TagF-Strep, pTrc-TagF^{GK}-Strep, pTrc-TagF^{DF}-Strep, pTrc-TagF^{DW}-Strep, pTrc-TagF^{SDR}-Strep, pTrc-TagF^{FD}-Strep, and pTrc-PppA.

To construct the plasmids for expressing proteins in *P. aeruginosa*, the gene of interest containing its RBS and ORF was cloned to be driven by a *lac* promoter on the broad host range vector pRL662 (7). The PCR products of *P. aeruginosa tagF*, *tagF-Strep*, $tagF^{GK}$*-Strep*, and $tagF^{SDR}$*-Strep* were amplified with primers described in Supplementary Table S2 and respectively digested by *Xho*I/*Xba*I, and cloned into the same sites of pRL662, which resulted in the plasmids pTagF^{Pa}, pTagF^{Pa}-Strep, $\mathrm{pTagF}^{Pa\text{-}GK}$-Strep, and $\mathrm{pTagF}^{Pa\text{-}SDR}$-Strep. The PCR product of *P. aeruginosa fha1-HA* was amplified with primers described in Supplementary Table S2 and cloned into pTrc200 with appropriate enzyme sites, which resulted in the plasmid pTrc-$\mathrm{Fha1}^{Pa}$-HA.

To construct the plasmid for protein expression in *E. coli*, the DNA fragment containing *tagF* 1-214 was PCR-amplified with primers described in Supplementary Table S2 and cloned into pET28a(+) to create the plasmid pET28a(+)-TagF 1-214.

For the constructs used for yeast two-hybrid, the *fha*, *tagF-pppA*, *tagF*, $tagF^{GK}$, $tagF^{DF}$,

tagF^{DW}, *tagF*^{SDR}, *tagF*^{FD}, *tagF*^{Pa}, *tagF*$^{Pa\text{-}GK}$, *tagF*$^{Pa\text{-}SDR}$,and *fha1*^{Pa} ORFs (without stop codon) were PCR-amplified with primers described in Supplementary Table S2 and respectively cloned into pGBKT7 or pGADT7 with appropriate enzyme sites to create the plasmids pGBKT7-TagF-PppA, pGBKT7-TagF, pGBKT7-TagF^{GK}, pGBKT7-TagF^{DF}, pGBKT7-TagF^{DW}, pGBKT7-TagF^{SDR}, pGBKT7-TagF^{FD}, pGBKT7-TagF^{Pa}, pGBKT7-TagF$^{Pa\text{-}GK}$, pGBKT7-TagF$^{Pa\text{-}SDR}$, pGBKT7-Fha1^{Pa}, pGADT7-Fha, pGADT7-TagF, pGADT7-TagF^{Pa}, and pGADT7-Fha1^{Pa}, respectively.

For the constructs used for bacterial two-hybrid, the *tagF*^{Pa} and *fha1*^{Pa} ORFs (without stop codon) were PCR-amplified with primers described in Supplementary Table S2 and respectively cloned into pKT25 (8) or pUT18C (8) with appropriate enzyme sites to create the plasmids pKT25-TagF^{Pa}, pKT25-Fha1^{Pa}, pUT18C-TagF^{Pa}, and pUT18C-Fha1^{Pa}, respectively.

References

1. Kado, C. I., and Heskett, M. G. (1970) Selective media for isolation of *Agrobacterium*, *Corynebacterium*, *Erwinia*, *Pseudomonas*, and *Xanthomonas*. *Phytopathology* **60**, 969-976
2. Bertani, G. (1951) Studies on lysogenesis. I. The mode of phage liberation by lysogenic Escherichia coli. *J Bacteriol* **62**, 293-300
3. Lossi, N. S., Manoli, E., Simpson, P., Jones, C., Hui, K., Dajani, R., Coulthurst, S. J., Freemont, P., and Filloux, A. (2012) The archetype Pseudomonas aeruginosa proteins TssB and TagJ form a novel subcomplex in the bacterial type VI secretion system. *Mol Microbiol* **86**, 437-456
4. Lin, J. S., Ma, L. S., and Lai, E. M. (2013) Systematic Dissection of the Agrobacterium Type VI Secretion System Reveals Machinery and Secreted Components for Subcomplex Formation. *PLoS One* **8**, e67647
5. Quandt, J., and Hynes, M. F. (1993) Versatile suicide vectors which allow direct selection for gene replacement in gram-negative bacteria. *Gene* **127**, 15-21
6. Schmidt-Eisenlohr, H., Domke, N., and Baron, C. (1999) TraC of IncN plasmid pKM101 associates with membranes and extracellular high-molecular-weight structures in Escherichia coli. *J Bacteriol* **181**, 5563-5571
7. Vergunst, A. C., Schrammeijer, B., den Dulk-Ras, A., de Vlaam, C. M., Regensburg-Tuink, T. J., and Hooykaas, P. J. (2000) VirB/D4-dependent protein translocation from Agrobacterium into plant cells. *Science* **290**, 979-982
8. Karimova, G., Pidoux, J., Ullmann, A., and Ladant, D. (1998) A bacterial

two-hybrid system based on a reconstituted signal transduction pathway. *Proc Natl Acad Sci U S A* **95**, 5752-5756
