# Supplementary material for: TagF-mediated repression of bacterial type VI secretion systems involves a direct interaction with the cytoplasmic protein Fha
Source: J Biol Chem. 2018 Mar 29;293(23):8829–42. doi: 10.1074/jbc.RA117.001618 (PMC5995506; doi:10.1074/jbc.RA117.001618)
Supplement: Supporting Information [file supp_RA117.001618_134892_1_supp_91529_p5bkyg.pdf]

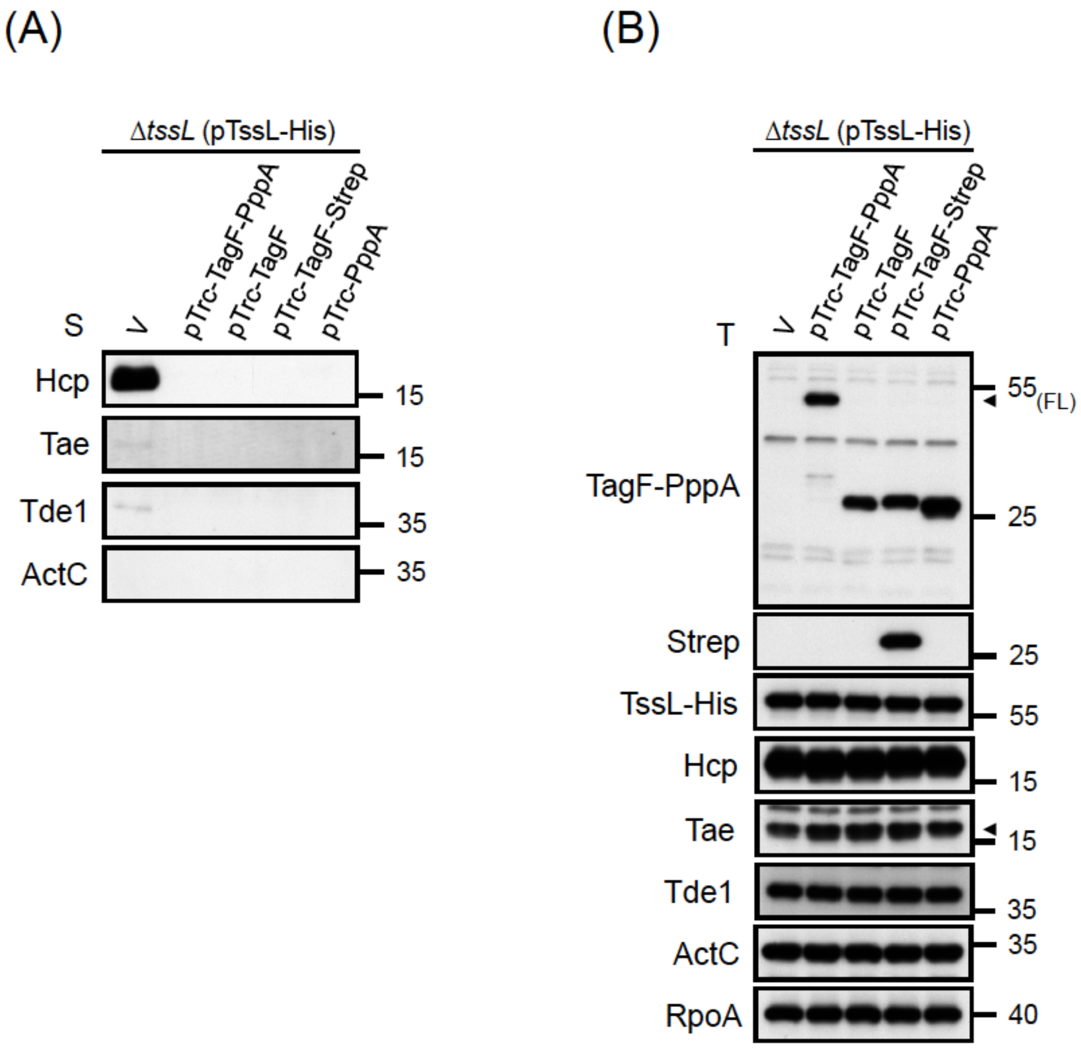

Figure S1 Type VI secretion assay in Δ*tssL* harboring two plasmids. Western blot analysis of secreted (S) **(A)** and total (T) **(B)** proteins isolated from Δ*tssL* (pTssL-His) harboring the vector pTrc200 (V) or *tagF-pppA*–overexpressing plasmid (pTrc-TagF-PppA) or *tagF*–overexpressing plasmid (pTrc-TagF) or *tagF-Strep*–overexpressing plasmid (pTrc-TagF-Strep) or *pppA*–overexpressing plasmid (pTrc-PppA) grown in AB-MES (pH 5.5) liquid culture with specific antibodies. The non-secreted protein ActC and RNA polymerase α subunit RpoA were internal controls. The proteins analyzed and molecular weight standards are on the left and right, respectively, and indicated with an arrowhead when necessary. FL, full-length TagF-PppA proteins.

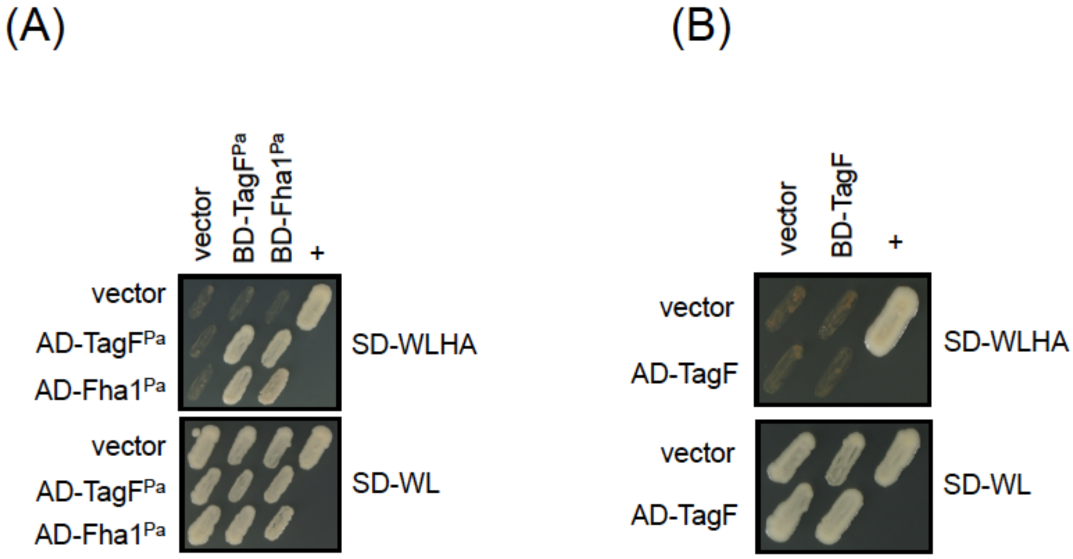

Figure S2 Yeast two-hybrid protein–protein interaction studies. Yeast two-hybrid protein–protein interaction results with **(A)** *P. aeruginosa* TagF ($TagF^{Pa}$) and Fha1 ($Fha1^{Pa}$) proteins and **(B)** *A. tumefaciens* TagF proteins. SD-WL medium (SD minimal medium lacking Trp and Leu) was used for selecting plasmids. SD-WLHA medium (SD minimal medium lacking Trp, Leu, His, and Ade) was used for auxotrophic selection of bait and prey protein interactions. The positive interaction was determined by growth on SD-WLHA medium at 30 ^{o}C for at least 2 days. The positive control (+) showing interactions of SV40 large T-antigen and murine p53 and negative control (vector) are indicated.

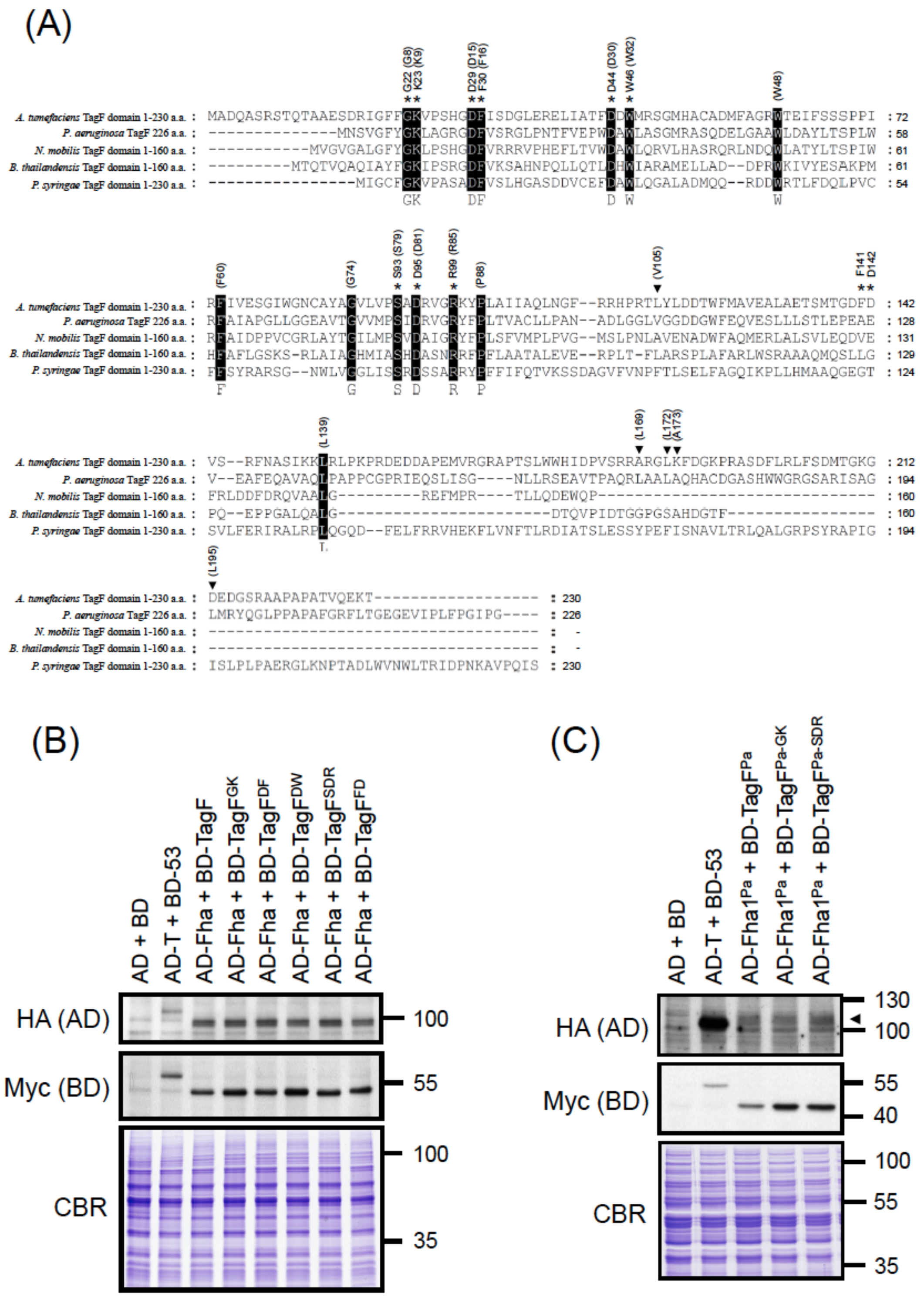

Figure S3 Amino acid sequence alignment of TagF orthologs, and western blot analysis of total proteins from yeast with various plasmid combinations. (A) Amino acid sequence alignment of the TagF or TagF domain orthologs from *A. tumefaciens* (TagF-PppA/Atu4331, accession: NP_356324.2), *P. aeruginosa* (TagF/PA0076, accession: NP_248766.1), *Nitrococcus mobilis* (NB231_12224, accession: ZP_01126757.1), *Burkholderia thailandensis* (Hypothetical protein BTH_I2955, accession: YP_443462.1), and *Pseudomonas syringae* (Hypothetical protein PSPPH_0124, accession: YP_272434.1). Conserved amino acid residues are highlighted in black and marked below, and G22, K23, D44, W46, S93, D95, R99,

F141 and D142 used for mutagenesis are indicated with an asterisk. The relative positions of these conserved amino acid residues in *P. aeruginosa* $TagF^{Pa}$ are respectively indicated in parentheses. The residues required for dimer formation in *P. aeruginosa* TagF (V105, L169, L172, A173, and L195 in $TagF^{Pa}$) (34) are indicated with a down arrow. Sequences were aligned and highlighted by use of ClustalW2 (http://www.ebi.ac.uk/Tools/msa/clustalw2/). Part of the aligned result is shown in Figure 6A. **(B)** According to Figure 6D. Western blot analysis of total (T) proteins isolated from yeast with various plasmid combinations with specific antibodies. The AD vector expressing SV40 large T-antigen (T) or *A. tumefaciens* Fha proteins tagged with HA, and BD vector expressing murine p53 (53) or various *A. tumefaciens* TagF (TagF, $TagF^{GK}$, $TagF^{DF}$, $TagF^{DW}$, $TagF^{SDR}$, and $TagF^{FD}$) proteins tagged with Myc. **(C)** According to Figure 8A. Western blot analysis of total (T) proteins isolated from yeast with various plasmid combinations with specific antibodies. The AD vector expressing SV40 large T-antigen (T) or *P. aeruginosa* Fha1 ($Fha1^{Pa}$) proteins tagged with HA, and BD vector–expressing murine p53 (53) or various *P. aeruginosa* TagF ($TagF^{Pa}$, $TagF^{Pa\text{-}GK}$, and $TagF^{Pa\text{-}SDR}$) proteins tagged with Myc. All protein samples were analyzed by SDS-PAGE followed by Coomassie blue staining (CBR) and served as an internal control. The proteins analyzed and molecular weight standards are on the left and right, respectively, and indicated with an arrowhead when necessary.

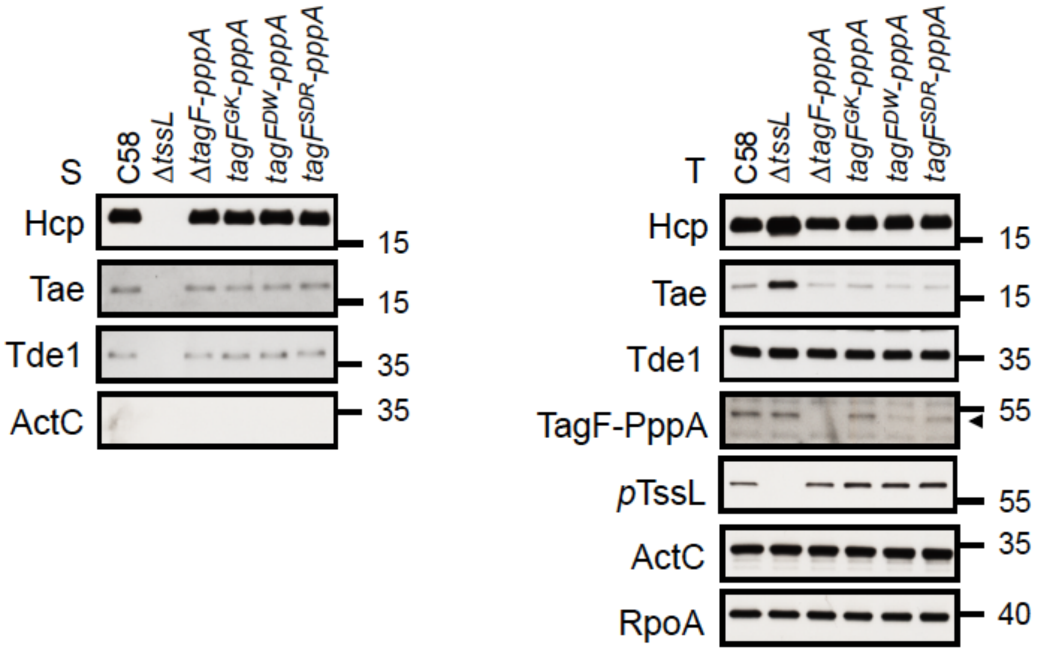

Figure S4 Type VI secretion analysis. Western blot analysis of total (T) and secreted (S) proteins isolated from wild-type C58, Δ*tssL,* Δ*tagF-pppA,* or chromosomally encoded *tagF-pppA* variants, including *tagF-pppA* with substitutions of *tagF* domain (*tagF^{GK}-pppA*, *tagF^{DW}-pppA*, and *tagF^{SDR}-pppA*) grown in AB-MES (pH 5.5) liquid culture with specific antibodies. The non-secreted protein ActC and RNA polymerase α subunit RpoA were internal controls. The proteins analyzed and sizes of molecular weight standards are on the left and right, respectively, and with arrow when necessary.
